# Supplementary material for: Influence of surprise on reinforcement learning in younger and older adults
Source: PLoS Comput Biol. 2024 Aug 14;20(8):e1012331. doi: 10.1371/journal.pcbi.1012331 (PMC11346965; doi:10.1371/journal.pcbi.1012331)
Supplement: S1 Text — Supplementary information file including additional analyses of parameter recovery, model recovery and the combined Valence and Surprise model. This file includes Fig A (Performance early in the task for each age group), Fig B (Parameter recovery), Fig C (Model recovery.) and Fig D (Posterior predictive check). Figure legends see inside S1 Text. (PDF) [file pcbi.1012331.s001.pdf]

Supplementary Material: Influence of surprise on reinforcement learning in younger and older adults

Christoph Koch[1,2,*], Ondrej Zika[1,3], Rasmus Bruckner[1,4], Nicolas W. Schuck[1,3,5,*]

[1] Max Planck Research Group NeuroCode, Max Planck Institute for Human Development, Berlin, Germany

[2] International Max Planck Research School on the Life Course, Max Planck Institute for Human Development, Berlin, Germany

[3] Max Planck UCL Centre for Computational Psychiatry and Aging Research, Berlin, Germany, and London, United Kingdom

[4] Department of Education and Psychology, Freie Universität Berlin, Berlin, Germany

[5] Institute of Psychology, Universität Hamburg, Hamburg, Germany

*Corresponding authors (koch@mpib-berlin.mpg.de, schuck@uni-hamburg.de)

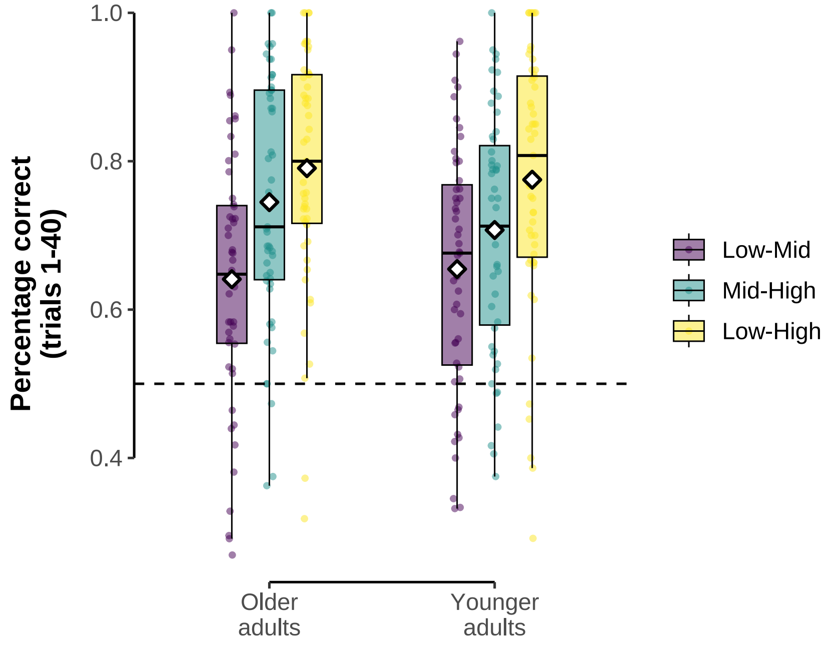

Figure A: Performance early in the task for each age group. The y-axis shows the percentage of correct free choices (i.e. choosing bandit with higher average outcome) in the first 40 trials within one run of the task. Age groups are shown on the x-axis. Data is displayed separately for each bandit combination (low-mid, mid-high, low-high, see colors/legend). Dots show values of individual participants and white diamonds show mean within each bandit combination and group. Dashed line indicates chance-level performance.

1 Parameter Recovery

To assess the integrity of our fitting process we performed parameter recovery on all candidate models. We simulated model choices by having each model play through the reward schedules of the reported sample according to their respective equations. For each simulation, the model parameters (α, π, α_{pos}, α_{neg}, l, s and u) were chosen randomly from a uniform distribution spanning the specified range for each parameter (see Methods in main text). The beta parameters of the outer loop regression were kept constant at the values of 0 (β_0, bias in choosing left or right bandit), -1 (β_1, β_3, i.e. value and uncertainty for left bandit), and 1 (β_2, β_4, i.e. value and uncertainty for right bandit) for each candidate model during the fitting process. This ensured direct influence of the parameters on the model's choices. The resulting model choices were then used for a model fitting procedure identical to that reported in the main text. As a measure for parameter recovery we here report the correlation between the input parameters and recovered parameters.

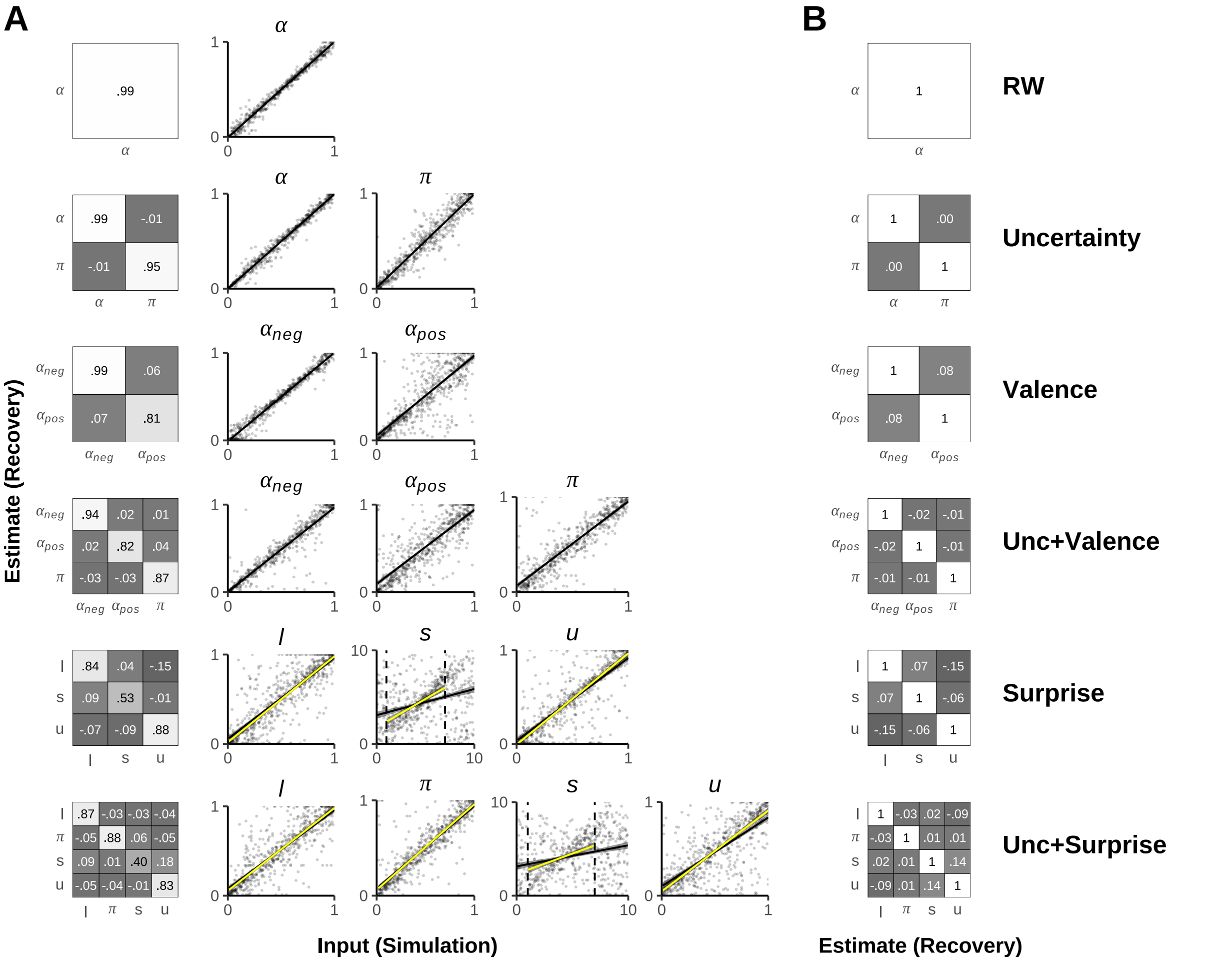

Figure B: (A): Correlation between input parameters used for simulation and parameters recovered from model fitting procedure on the simulated data. Each row displays results for a different model with names shown on the far right. Correlation matrix on the left shows Spearman correlation between input parameters and recovered parameters. Strong correlation is indicated by lighter colors. Higher values on the diagonal (top-left to bottom-right) suggest better parameter recovery for the respective model. Correlation plots on the right display the relationship between input parameter and recovered parameter for the diagonal of the matrix. Models including the parameter s (*Surprise* and *Surprise+Unc*) display an additional line (yellow) that displays the relationship when s was constrained between 1 and 7, as recovery for values outside this range was poor. In these cases the correlation matrix (left) shows the correlation for $s \in [1, 7]$. (B): Correlation matrices (Spearman) between recovered parameters for each model. Strong correlation indicated by lighter colors. Apart from the diagonal, high values suggest the parameters of a model are trading off against each other.

The results are displayed in Fig. BA. The parameters of the *Rescorla-Wagner* (RW) model and the *Valence* model recovered well, showing correlations between input and recovered parameters of $r \geq .81$. Similarly high values were achieved for the parameters l and u in the *Surprise* model. For the initially planned interval between 0 and 10 the s parameter of the *Surprise* model recovered poorly. We therefore constrained s to lie between 1 and 7 as predominantly values outside of this range showed poor recovery (see *Surprise* model in Fig. BA). This constrained interval led to recovery of the s parameter with $r = .53$. The *Uncertainty* model showed good

recovery of the α parameter ($r = .99$) and the π parameter ($r = .95$). The parameters in the combined models showed similar recovery to the other candidate models which did not include an uncertainty component. One exception was the s parameter in the *Unc+Surprise* model, which showed the lowest correlation at $r = .40$.

We furthermore looked at the correlation between recovered parameters in each model (see Fig. BB) to identify parameters that systematically trade-off against each other. The strongest, yet relatively weak correlation between estimated parameters was found in the *Surprise* model between the l and u parameters ($r = -.15$). This suggested a light trade-off between both parameters in the model fitting process.

2 Model recovery

Following the same process as for parameter recovery, we also extracted the best fitting model for each simulated data set. The best fitting model was given by the model with the lowest AICc after the model fitting process. The recoverability of each model was given by the percentage the model fitting process determined the same model the best fitting model that was also used to simulate the data. Results of the model recovery are shown in figure C. Out of the models without an uncertainty component the *RW* model showed the highest recovery rate of 57%, followed by the *Valence* (46%) and the *Surprise* (44%) models. For the *Valence* and *Surprise* models a frequent false recovery was the *RW* model (24% and 26%, respectively). This could be because certain parameter combinations of the *Valence* ($\alpha_{\text{pos}} \approx \alpha_{\text{neg}}$) and *Surprise* ($l \approx u$) models will cause effectively equal choices to a *RW* model with a single learning rate. Together with the appropriate penalization for the number of parameters of the AICc measure this could lead to a higher frequency of the *RW* model winning the model comparison during recovery.

The uncertainty model showed the highest recovery rate out of all models at 82%. However, the combined models' recovery was problematic and favored the simpler formulation of the *Uncertainty* model over the more complex *Unc+Valence* and *Unc+Surprise* models.

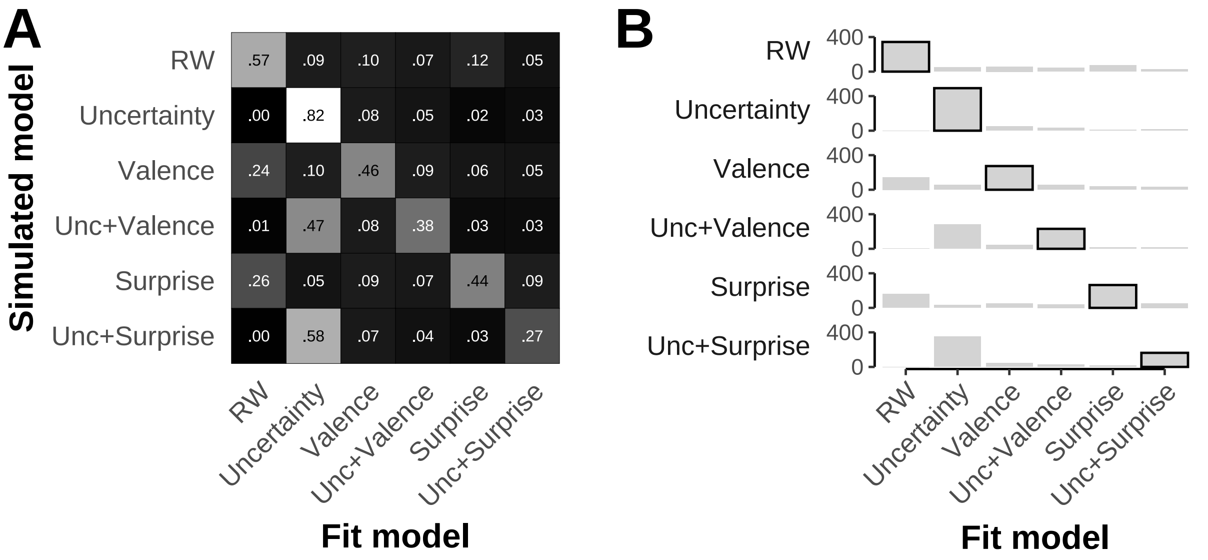

Figure C: Model recovery. (A): Confusion matrix of model recovery process. Plot shows the relative proportion with which each candidate model offered the best fit (x-axis), given a certain model was used for simulation (y-axis). Brighter colors indicate higher proportions. For visibility, numbers on a dark background are depicted in white. Ideal model recovery would produce an identity matrix. (B): Histogram of model recovery process. Plot shows the absolute number of times which candidate model offered the best fit (x-axis), given a certain model was used for simulation (y-axis). Bars showing matching models for simulation and fit are highlighted with a black outline. Note this panel is just a depiction of the absolute counts during model recovery. The same information expressed in relative terms is shown in panel A.

While out of the investigated models our findings and the posterior predictive check still speak in favor of the *Surprise* model, using the present model recovery also showed the fit between experiment and candidate models can be improved upon. As mentioned in the discussion, one core problem when trying to model the effect of highly surprising events on choices in stationary environments is that these highly surprising events cannot happen too often during the experiment. This limits the amount of critical trials in which models can strongly diverge in their predictions which might not be picked up by the log-likelihood across trials and hindering their recovery. We tried to address this issue by focusing the model fitting process to choices between the low and mid bandit (see Methods in main text). Additionally, this highlights the importance of posterior predictive checks, that can investigate differences between models on critical trials, even if their choices are similar over large proportions of the experiment. As shown in the Results in the main text, the *Surprise* model offered the closest resemblance to participants' data in such a posterior predictive check.

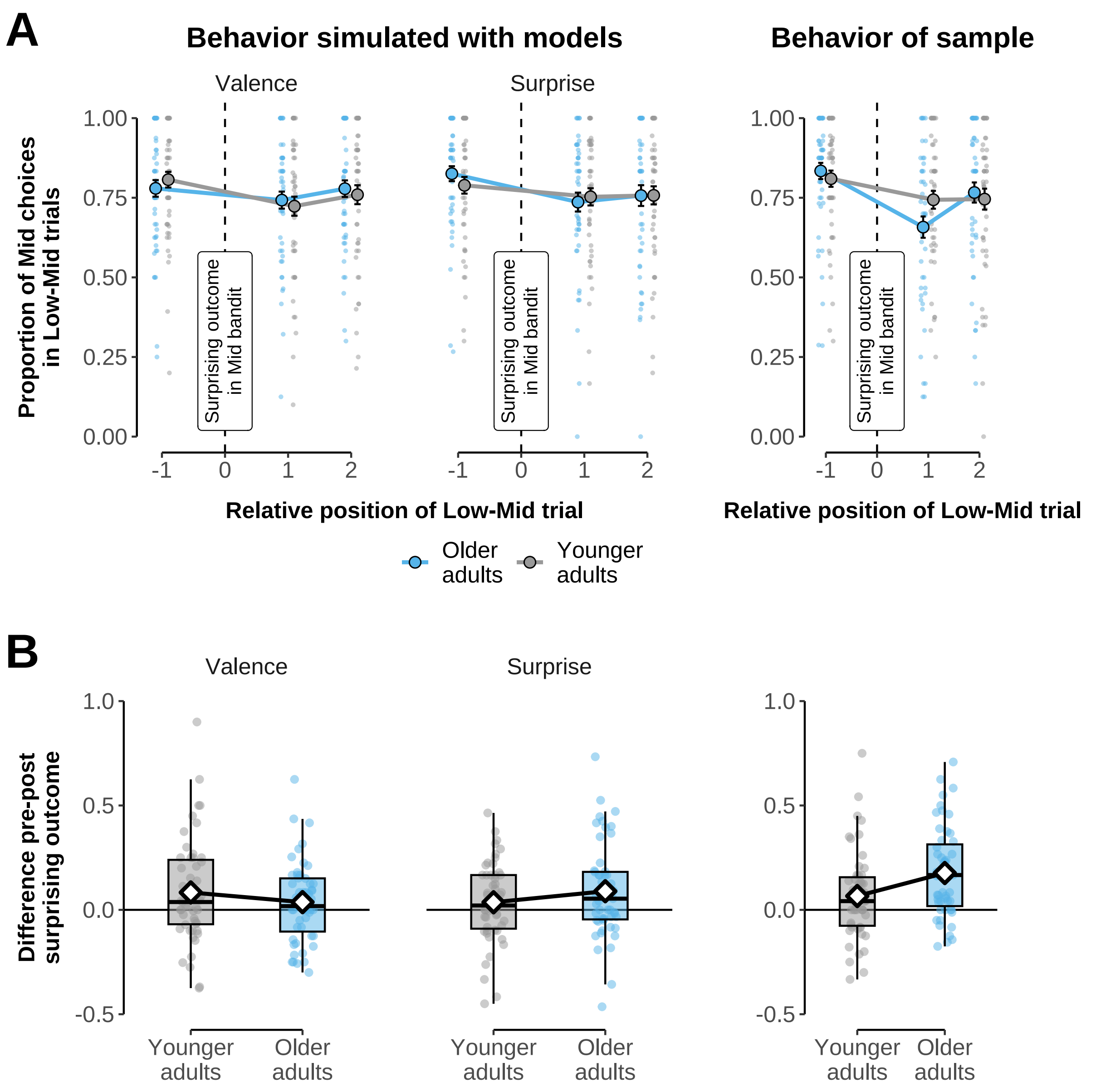

Figure D: Posterior predictive check. (A): Influence of surprising outcomes on choices in Low-Mid trials in choices simulated from candidate models (left) and real sample (right, identical to Fig. 3A in the main text). For each model a data set was simulated by using the parameter fits of each participant. The simulated data sets of each model are compared to the real sample to assess the model's capability of showing central behavioral effects. Analogous to Fig. 3A in the main text the plot shows the proportion of mid bandit choices in low-mid trials one trial before and two trials after participants experienced a surprising outcome from the mid bandit (vertical dashed line). Data shown separately for older (blue) and younger adults (grey). Each small dot is one participant, large dots depict group means with standard error of the mean shown by error bars. (B): Difference between proportion of mid bandit choices in low-mid trials pre and post a surprising outcome in the mid bandit for both age groups. As in panel A the plot is shown for choices simulated from candidate models (left) and the real sample (right). Higher values indicate a stronger reduction in choosing the Mid bandit immediately after a surprising outcome (pre − post). Dots show individual values of participants and diamonds show group means. The line connecting the group means was added as a visual aid to easier follow the direction of age differences.

3 Combined model: Valence and Surprise

In a final model we addressed the question if learning from outcomes lower than one's expectation was differently impacted by surprise in comparison to learning from outcomes higher than one's expectation. The resulting Valence+Surprise model followed the principle of the Surprise model but included two sets of l, u, and s parameters which separately defined a mapping between learning rates and positive or negative prediction errors (see Fig. 4D in main article for examples of this mapping).

$$V_{k,t+1} = V_{k,t} + \begin{cases} \left(l_{\text{pos}} + \dfrac{2}{1 + \widehat{\text{PE}}^{-s_{\text{pos}}}} \left(u_{\text{pos}} - l_{\text{pos}}\right) \right) \text{PE}_t, & \text{if PE} \geq 0 \\ \left(l_{\text{neg}} + \dfrac{2}{1 + \widehat{\text{PE}}^{-s_{\text{neg}}}} \left(u_{\text{neg}} - l_{\text{neg}}\right) \right) \text{PE}_t, & \text{if PE} < 0 \end{cases} \tag{S1}$$

Akin to the RW, Valence, and Surprise model, the probability to chose bandit k over bandit l was given by a logistic regression that included an intercept as well as the influence of the values of bandit k and l (see Eqn. 8 in the main article).

An AICc-based model comparison as described in the main article's methods section that included the Valence+Suprise model showed no major changes to the results reported in the main article. The Surprise model had the highest protected exceedance probability (72.54%), followed by the Valence model (27.21%). The Valence+Surprise model offered the best fit for the behavior of five participants across both age groups (3 younger adults, 2 older adults).

Importantly, the model recovery process based on AICc showed that the Valence+Surprise model could not be recovered from behavior simulated using the model. The model recovered successfully in only 2% of the simulated datasets. Instead, the data simulated using the Valence+Surprise model was best explained by the Surprise model (53%) not accounting for prediction error valence, the RW model (18%), or the Unc+Surprise model (11%).

List of Legends

Fig A. Performance early in the task for each age group. The y-axis shows the percentage of correct free choices (i.e. choosing bandit with higher average outcome) in the first 40 trials within one run of the task. Age groups are shown on the x-axis. Data is displayed separately for each bandit combination (low-mid, mid-high, low-high, see colors/legend). Dots show values of individual participants and white diamonds show mean within each bandit combination and group. Dashed line indicates chance-level performance.

Fig B. Parameter recovery. A: Correlation between input parameters used for simulation and parameters recovered from model fitting procedure on the simulated data. Each row displays results for a different model with names shown on the far right. Correlation matrix on the left shows Spearman correlation between input parameters and recovered parameters Strong correlation is indicated by lighter colors. Higher values on the diagonal (top-left to bottom-right) suggest better parameter recovery for the respective model. Correlation plots on the right display the relationship between input parameter and recovered parameter for the diagonal of the matrix. Models including the parameter s (*Surprise* and *Surprise+Unc*) display an additional line (yellow) that displays the relationship when s was constrained between 1 and 7, as recovery for values outside this range was poor. In these cases the correlation matrix (left) shows the correlation for $s \in [1, 7]$. B: Correlation matrices (Spearman) between recovered parameters for each model. Strong correlation indicated by lighter colors. Apart from the diagonal, high values suggest the parameters of a model are trading off against each other.

Fig C. Model recovery. A: Confusion matrix of model recovery process. Plot shows the relative proportion with which each candidate model offered the best fit (x-axis), given a certain model was used for simulation (y-axis). Brighter colors indicate higher proportions. For visibility, numbers on a dark background are depicted in white. Ideal model recovery would produce an identity matrix. B: Histogram of model recovery process. Plot shows the absolute

number of times which candidate model offered the best fit (x-axis), given a certain model was used for simulation (y-axis). Bars showing matching models for simulation and fit are highlighted with a black outline. Note this panel is just a depiction of the absolute counts during model recovery. The same information expressed in relative terms is shown in panel A.

Fig D. Posterior predictive check. A: Influence of surprising outcomes on choices in Low-Mid trials in choices simulated from candidate models (left) and real sample (right, identical to Fig. 3A in the main text). For each model a data set was simulated by using the parameter fits of each participant. The simulated data sets of each model are compared to the real sample to assess the model's capability of showing central behavioral effects. Analogous to Fig. 3A in the main text the plot shows the proportion of mid bandit choices in low-mid trials one trial before and two trials after participants experienced a surprising outcome from the mid bandit (vertical dashed line). Data shown separately for older (blue) and younger adults (grey). Each small dot is one participant, large dots depict group means with standard error of the mean shown by error bars. B: Difference between proportion of mid bandit choices in low-mid trials pre and post a surprising outcome in the mid bandit for both age groups. As in panel A the plot is shown for choices simulated from candidate models (left) and the real sample (right). Higher values indicate a stronger reduction in choosing the Mid bandit immediately after a surprising outcome (pre − post). Dots show individual values of participants and diamonds show group means. The line connecting the group means was added as a visual aid to easier follow the direction of age differences.
