# Supplementary material for: Influence of surprise on reinforcement learning in younger and older adults
Source: PLoS Comput Biol. 2024 Aug 14;20(8):e1012331. doi: 10.1371/journal.pcbi.1012331 (PMC11346965; doi:10.1371/journal.pcbi.1012331)
Supplement: Attachment — Submitted filename: PointByPoint_Kochetal_Resubmission.pdf [file pcbi.1012331.s003.pdf]

We thank both reviewers for their excellent comments. In our point-by-point reply below we will first repeat each reviewer comment in a box, and then add our reply below. Quoted text from the manuscript is indented and italics, and new text within these quotes is highlighted in yellow.

Reviewer 1

Reviewer Summary

In this paper by Koch et al., the authors employ a two armed bandit task with Gaussian rewards in combination with computational modeling to test the hypothesis that older adults are more sensitive to surprising outcomes when making decisions under uncertainty. The behavioral task is designed to decouple surprise, the main variable of interest, from reward magnitude, probability and uncertainty. The main claim of the paper is that older adults are more sensitive to surprising events than younger adults. The behavioral analysis reveals interesting age-related behavioral effects that support the claim, but are not recapitulated by the computational modeling analysis. I found the task design and analysis to be careful, and the paper balanced in its claims. The main weakness is in the discrepancy between behavioral and computational modeling results. Below are some comments and suggestions that may address this issue.

Major Comments

R1C01: The bimodal distribution of the mid-bandit results in predominately negative large prediction errors – so taking the absolute value and saying the surprise is “valence free” is not truly accurate as a majority of these are actually negative surprise. Why wasn’t an additional condition included that induced large predominately positive PEs?

We thank the reviewer for their comment and question. We are happy to outline our reasoning that led us to this design, which we believe offers unique advantages, even after taking the limitations noted by the reviewer into account.
Our focus was to observe choices after participants experienced a surprising outcome. A main realization was that while surprising outcomes from the mid bandit alter participants’ value estimates, any subsequent choices of the mid bandit will likely be followed by a non-surprising outcome, and hence soon revert these changes. To make our effect as strong and long-lived as possible, we therefore tried to find a design in which participants would not immediately choose the mid bandit again after a surprise trial. This is naturally truer when the surprises are tied to negative prediction errors, since these lower a bandit's value estimate, and hence make it less likely to be chosen immediately afterward. The opposite is true for large positive prediction errors, which lead to more mid bandit choices afterwards, thereby quickly canceling out any

changes in value and their possible effects on behavior that were the focus of our investigation. In addition, the negative prediction error design also had the advantage that the core prediction would be choices of the lowest bandit, which had a low baseline and represent a quite compelling case in our opinion. Unfortunately, our piloting revealed that data quality quickly declines the longer an experiment gets, and therefore prevented us from implementing a within-subject design that would have allowed us to compare both conditions.

These considerations aside, we also implemented a model that is sensitive to PE magnitude while simultaneously differentiating between positive and negative prediction errors, as suggested by the reviewer in R1C02 below (Valence+Surprise Model). We found that the surprise model explained participants' behavior better. We discuss these results, and how they affect our manuscript below. We hope that thereby we could address the reviewer's concern.

R1C02: Relatedly, have the authors considered a way to combine the "Valence" and "Surprise" model? It is possible that learning from negative outcomes might be impacted differently by surprise than learning from positive outcomes.

Both reviewers have suggested this change, and we thank both of them for this contribution, which we are happy to implement. This combined model includes the key feature of the Surprise model, a mapping between learning rate and magnitude of prediction error (surprise), but also allows learning rates to be different for positive vs negative prediction errors.

Fitting this model to our data showed that it had an overall higher AICc than many of the other models, and that only a small number of subjects were fit best by it, as shown in Figure R1 below. In addition, we found that the combined Valence+Surprise model had poor model recovery: data simulated using the Valence+Surprise model was only correctly assigned to the Valence+Surprise model in 3% of the cases, which was notably lower than the recovery rate observed in all other models (see Figure R2 below). In most cases, the recovered model was the simpler Surprise model (50%) which does not account for prediction error valence.

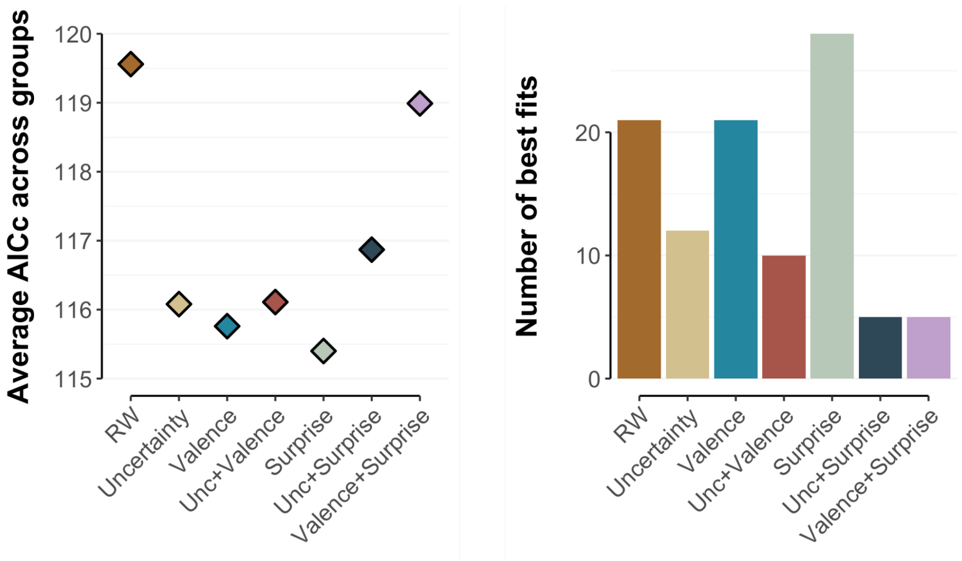

Figure R1. Left: Total average AICc values for the novel Valence+Surprise model in comparison to all other models. Right: number of participants best fit by the novel Valence+Surprise model in comparison to all other models.

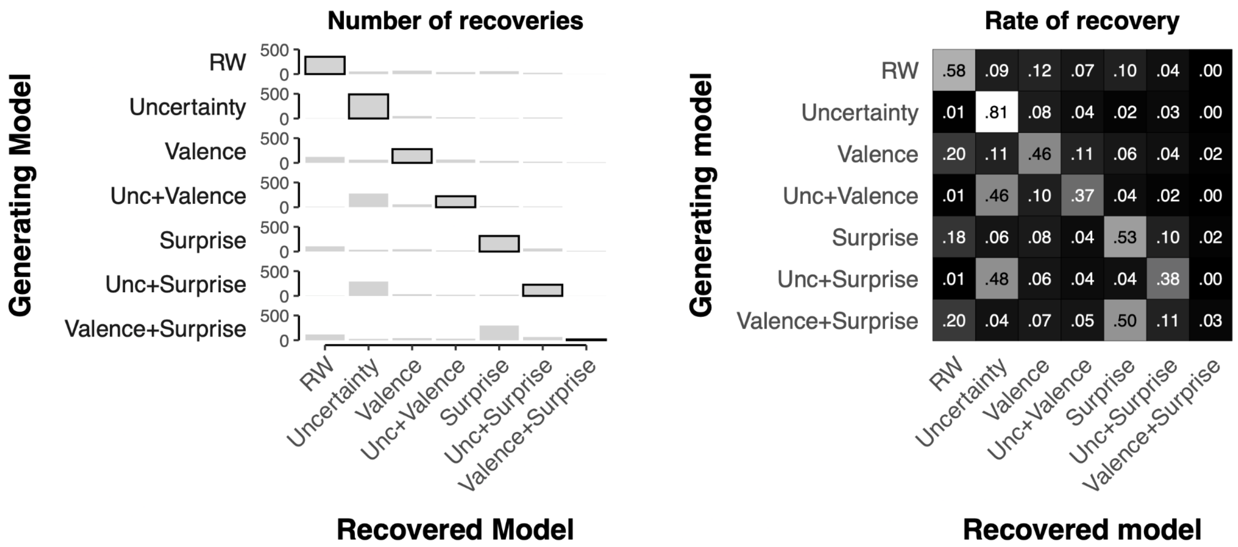

Figure R2. Model recovery including the novel Valence+Surprise model (bottom most model), in comparison to all other models. Left plot shows the absolute number of recoveries, the right plot shows relative rates of recovery. The combined model had notably poor recovery.

Given that the Surprise+Valence model did not provide a compelling account of behavior, in combination with the nature of our design that is not ideal to test such a model, we decided to report information about the new Surprise+Valence model mostly in the SI of the manuscript, while adding the following notes in the main text that inform the reader of the additional model and its results:

Ll. 695-699 (Methods):

"We also implemented an additional model that combined the functionality of the Surprise model with that of the Valence model potentially capturing if surprise from positive or negative prediction errors differently affects learning. This model did not provide any superior fits compared to the winning models reported here. Details can be found in the SI."

Ll. 279-281 (Results):

"Results of an additional model that combined the functionality of the Surprise and Valence models can be found in the SI."

R1C03: The "Valence" model has some additional machinery to handle the large negative surprises that the RW model does not, which explains why it also captures the behavioral effect of interest. It is not obvious what additional machinery the "Surprise" model offers, especially given that there is only one qualitative kind of surprise in the mid-bandit. Some simulation work might help elucidate how this model's predictions are different from the "Valence" model.

We appreciate the reviewer's comment that the nature of our model was not entirely clear. To clarify the major aspects of our model, we now inserted the following statement into the manuscript's results section (ll. 256-264):

"*The core idea of this model, compared to the Rescorla-Wagner and Uncertainty models, was that how much change in value results from a particular outcome depends on the absolute prediction error. The model was designed to incorporate various relationships between absolute prediction error and learning rate, including both higher learning rates for low prediction errors and the opposite. Moreover, we assumed that the effect of prediction error on learning rate is instantaneous, i.e. affects updating on the trial immediately, in contrast to the Uncertainty model, where prediction errors on trial t only come to influence the learning rate on trial t+1.*"

Furthermore, we have included posterior predictive checks to investigate how these different mechanisms translate into predictions of behavior. We mention these in the main manuscript (ll. 303-308):

"*We next performed posterior predictive checks to ask whether the two models with the highest protected exceedance probability (Surprise and Valence model) showed the main behavioral observation of interest, i.e. the outsized effect of large absolute PE events on choices (see Methods). We used the estimated models to generate synthetic data for each model. We then analyzed the generated data sets in an identical manner to the participant's data. A graphical comparison can be found in Figure S4 in the supplement.*"

The mentioned Figure is reproduced below, and the results of the posterior predictive checks are reported in more detail in the main manuscript in ll. 300-313.

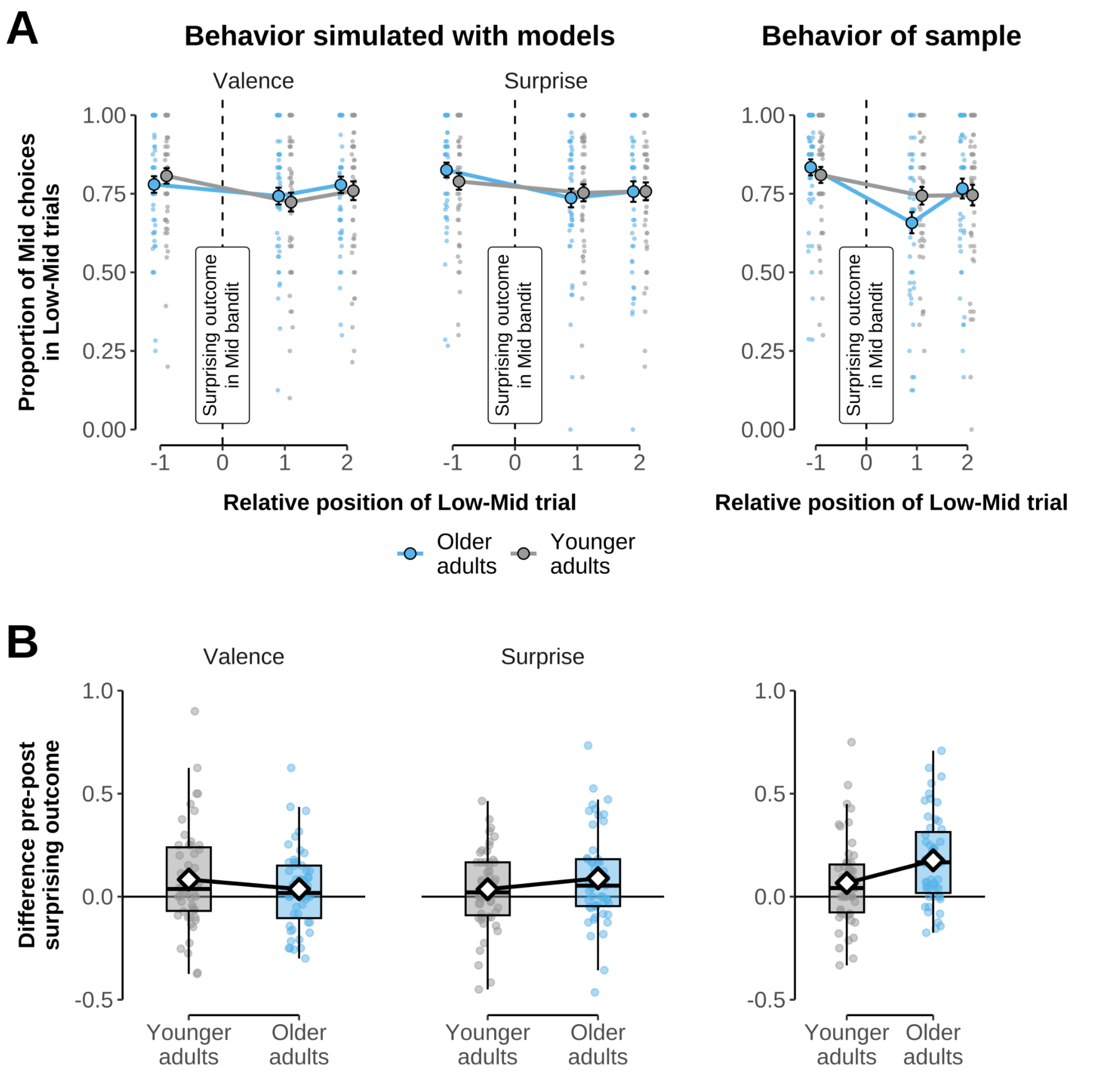

In short, the posterior predictive checks show that both with the fitted parameters, the Surprise model can replicate a lower probability to choose the mid bandit in low-mid bandit trials after a surprising outcome was obtained in older adults. The Valence model predicts a weaker change overall and does not appear to capture the directionality of the effect across age groups.
As we note in the manuscript, the magnitude of this effect is clearly smaller than what we observed in the data. Further checks of running the model under hand-picked parameter combinations showed that it is in principle capable of producing a stronger pattern. Hence, it appears that the resultant fitted parameters reflect a tradeoff between the model's attempt to fit the pattern of behavior in large surprise trials and all other trials. This is an important issue for future research that we now also mention in the discussion (ll. 452-459):

“*One limitation of this work was the fact that model parameters did not reflect age differences evident in behavioral analyses. Specifically, the model did not suggest a heightened sensitivity to surprising outcomes that is more pronounced in older adults. A potential reason for this finding might be that the effects of surprising outcomes on participants’ choices can only be reflected in a limited number of trials, reflecting a problem inherent in the study of surprising events. This holds the potential danger of model fits that are largely dominated by behavior in which the differential effects of surprise cannot be reflected in participants' choices.*”

We hope the changes in the manuscript help to address this important issue and provide sufficient information to make the differences between the Valence and Surprise model clearer to the reader.

#R1C04: I assume that the models were fit in the same way to all the data, such that surprises in the high/low bandits are treated the same as surprises in the mid bandit, and the same u/l/s parameters apply to all three. Is it possible that some scaling issues might be at play here? This could be tested by separating u/l/s by whether the distribution was bimodal or not.

The reviewer raises an important point regarding differences between bandits. We agree with the idea that parameters of the surprise model that fit one bandit well might not apply to other bandits, for instance because events with the same absolute prediction error magnitude can have different probabilities in the different bandits.
For this reason, we have restricted the fitting to trials in which participants decide between low and mid trials. The models of course still experience a complete learning history, i.e. they observe the entire sequence of outcomes from all bandits, but the loss function of the parameter fitting was defined solely by the model’s ability to capture low-mid trials. This ensured that the results in overall fit and parameters did not suffer from the complications raised here.

We apologize that this important aspect of our paper has not become clear. We now underline this important aspect of the fitting process in the respective results section (ll. 226-232):

“*Building on these behavioral results, we used computational modeling to specifically contrast the contributions of surprise, uncertainty and differential learning from positive and negative prediction errors (as well as combination of these) to behavior. (...) We therefore modeled participants choices specifically in low-mid trials (see Methods) using the following four main and two combination models:*”

In addition, we have now added the following sentences to the methods section (ll. 711-713):

"Importantly, all models were fit solely to participants' free choices in low-mid bandit comparisons. This was done to specifically capture the behavioral effect in response to the one-sided bimodal distribution of the mid bandit."

R1C05: The main hypotheses are not explicitly stated - e.g. the introduction expanded enough on specific hypotheses regarding age and surprise-based learning. There are two parallel goals in this study: (1) impact of surprise on RL and (2) the impact of age on this relationship. The rationale and implications of these related aims is not fully developed.

We thank the reviewer for pointing out that the hypotheses were stated not clearly enough in our previous manuscript. We have updated the introduction to state them more clearly now (ll. 74-95, most relevant sections are marked in bold):

"Other work in the domain of decisions from description suggests that older adults overweight low probability events in the gain domain (i.e., show more risk-seeking behavior), compared to younger adults (Pachur et al., 2017). This work has focused purely on how the stated probability of events affects decision making. In contrast, when decisions are based on learned probabilities, referred to as decisions from experience (Hertwig & Erev, 2009; Wulff, Mergenthaler-Canseco, & Hertwig, 2018), age-related differences in choice behavior and risk-taking often differ compared to decisions from description, where age differences in risk preferences depend on the exact choice scenario (Mata et al., 2011).
Therefore, we aimed to examine age-related learning and decision making differences in an experience-based choice task with stationary outcome probabilities. We specifically studied the effects of outcomes that are highly surprising, i.e. differ significantly from most previous outcomes. ***We stipulated that surprise could affect the learning rate with which participants update their expectations in a trial and error setting, even when dissociated from the effects of probability. In line with previous work, we expected that surprise would have a greater effect on older adults, as compared to younger adults.***
Taking a reinforcement learning (RL) perspective (Sutton & Barto, 2018; Dayan & Daw, 2008), we conceptualized surprise as the absolute prediction error (PE), i.e. the deviation of an observed outcome from the current expectation. ***While standard RL theory assumes that prediction errors are weighted by a constant learning rate parameter $\alpha \in [0, 1]$, we hypothesized that learning rates are modulated by the absolute PE, i.e. surprise of a given trial. Our idea specifically predicts that surprise impacts learning immediately, i.e. affects the update on the very same trial that caused the surprise.****"*

R1C06: In the key behavioral analysis comparing choice of mid vs. low bandit, did the authors always condition on whether the previous trial's outcome was from the dominant mode? Otherwise it is possible to have several "surprising" trials in a row, which might induce repetition effects, potentially explaining the lack of a result in the modeling (as consecutive surprise trials are treated the same).

We thank the reviewer for this important comment. The mentioned analysis did in fact not condition on previous trial history in the way mentioned by the reviewer. Note that there are two ways to define what “previous trial” means in the context of our experiment, and to avoid confusion we would like to provide information for both. For one, the previous trial can describe the trial that happened right before an outcome from the non-dominant mode, irrespective of which bandit was chosen. Regarding this interpretation, outcomes from the non-dominant mode happening in consecutive trials were very rare. On average, this was experienced in only 5.79% of trials that entered the behavioral surprise analysis for each participant. Alternatively, the previous trial can describe the last trial in which the mid bandit was chosen, meaning the last experienced outcome of the mid bandit, irrespective of what outcomes other bandits produced in between. Defined as in this way, we had an average of 22.60% of surprise repetition trials in our previous analysis (with “repetitions” separated by 2.87 trials in which other bandits were chosen). We assume this is what the reviewer was referring to when they raised the valid point of potential repetition effects on choices given participants experienced outcomes from the non-dominant mode in consecutive trials.
To investigate this question, we repeated the analysis of free choices in low-mid trials before and after participants encountered an outcome from the non-dominant mode after excluding the above mentioned 22.60% of trials. These results for this analysis were congruent with those reported in the main manuscript. The linear mixed model of the immediate effect of surprising outcomes showed significant main effects of position ($\chi^2(1) = 37.86$, $p < .001$) and of run ($\chi^2(1) = 10.27$, $p = .001$), both of which were reported if no trials are excluded from the analysis. The position X age interaction effect reported in the main manuscript was also qualitatively present in the results, albeit narrowly failing to surpass significance ($\chi^2(1) = 2.59$, $p = .107$). While this is a rather minor change in the pattern of results, it *could* suggest that the sensitivity to outcomes from the second mode accumulates somewhat over repetitions. At the same time, our modeling showed that an uncertainty model, which would capture only such cumulative effects, is not a good account of overall behavior. Hence, while investigating such repetition effects are interesting, we believe that the overall pattern of the results does not suggest that they play a major role in our study.

R1C07: Were participants aware of the distribution structure? How was the feedback schedule conveyed to them? The language used for instruction may impact participants’ overall surprise and uncertainty.

Since the data was collected online, instructions were delivered to all participants in a set of screens that included text and examples for all trial types of the task. At the bottom of this answer, we cite the exact instruction text. Neither instructions nor training included any information on the distributions of the bandits. Participants were only told that outcomes of the same bandit may vary and that their goal will be to collect as many points as possible (see highlighted text in the cited instructions). We are therefore confident that the instructions and training did not introduce a systematic bias in participants' expectations of surprise and uncertainty.
To also convey this information to the reader, we added the following sentences to the manuscript's methods section (ll. 534-540):

> "*Each bandit was indicated by a different Japanese Hiragana symbol (randomly assigned across participants). Participants had to learn about each bandits' value through trial and error and did not receive any information about reward distributions or reward schedules besides the obtained points. Points collected were translated into a monetary bonus of up to 3 GBP at the end of the experiment. Prior to the task all participants went through identical, text-based instructions and a short training period that conveyed information about the different trial types (see below), but not the differences in the underlying distributions.*"

The exact text displayed to all participants during the instruction and training of the task can be found below (pictures omitted). The section relevant to the reviewer's comment is marked in bold.

> "*In this study we examine how humans make reward-based decisions. To this end, we developed a task in which you will have to make choices and collect as many points as possible. We will now start a TRAINING that will show you how the task works. Remember that this is just the training! Any points you collect during the training WILL NOT COUNT TOWARDS YOUR FINAL SCORE and therefore not your bonus payment! For now, we just want to teach you what to do! First, we would like to ask you to place your LEFT INDEX FINGER on the F key on your keyboard and your RIGHT INDEX FINGER on the J key. Please press F or J on your keyboard to continue.*
> *During the task, you will have to choose between TWO AVAILABLE OPTIONS which are SEPARATED BY A CROSS. There are THREE OPTIONS OVERALL, but you will always be asked to choose between two at the time. In this training the three options will be represented by the letters A, B, and C. Later in the REAL EXPERIMENT the options will be represented by DIFFERENT SYMBOLS! For this training it will look similar to what you see below: (example of choice screen). Here you have an OPTION A (on the left side) and an OPTION B (on the right side). The cross in the middle will always stay on screen so you can focus on it with your eyes. You will have to SELECT one of these two options by pressing the KEY ON THE RESPECTIVE SIDE: Left index finger, F: Select left option (here A). Right index finger, J: Select right option (here B). Please press F or J on your keyboard to continue.*
> ***Every time you make a CHOICE you will be REWARDED WITH POINTS. We will***

immediately show you how many points you got for your choice. You will get BETWEEN 0 AND 100 POINTS for the option you selected. You will NEVER see how many points the other option would have given you. The amount of points you get from the same option will VARY! Your goal will be to find out which of the available options to choose to get the most points possible. *You have 3 SECONDS to make a choice. If you should take longer than 3 seconds YOU WILL NOT GET ANY POINTS for your choice so please answer within the 3 seconds. If you took longer than 3 seconds we will show this to you with a 'Please respond faster' prompt. The more points you will get the more pay you will receive so try to get a high score! The bonus payment will range between 0 and 3 GBP based on the amount of points you collect. We will now go through some examples. Please press F or J on your keyboard to continue and play through some examples.*

Good job! What you just did will be the main part of the task. However, sometimes you will encounter a FRAME around an option. This means that you HAVE TO CHOOSE the FRAMED OPTION. If you correctly choose the framed option, you will get the points for the framed option. If you chose the option without the frame you will NOT GET ANY POINTS so remember to choose the framed option. Also for these choices you have a time limit of 3 SECONDS. If you take too long for a choice you will not get any points and we will let you know with a 'Please respond faster' prompt. Let's go through some examples in the next screen. Remember that this is JUST THE TRAINING, so feel free to make a wrong choice on purpose! Please press F or J on your keyboard to continue any play through some examples.

Finally, we will sometimes ask you WHAT YOU THINK THE SCORE WOULD BE if you would choose a certain option. We will show one option to you together with two sliders. Number 1 is to tell us how many points you think you would get from this option. Number 2 is to tell us how much you think it might vary. You can USE YOUR MOUSE to move the sliders. For both sliders we are asking for a rough estimate, so don't be too concerned about a few points in accuracy. If we think you are taking too long we will let you know. Please remember that there are no right or wrong answers and you CANNOT EARN ANY POINTS with your estimation! We still ask you to be as accurate as possible. Let's try it out with a few examples! Please press F or J on your keyboard to continue with some examples.

From time to time you will get the option to TAKE A SHORT BREAK. You will recognize these breaks by this symbol and a text telling you about the break. Feel free to rest your hands and eyes during these breaks. You can continue with the task whenever you are ready by pressing F or J. During these breaks a countdown of 2 minutes will appear after which the experiment will continue automatically. You don't need to wait the full 2 minutes. Just continue whenever you feel ready. Please press F or J on your keyboard to continue.

In the real experiment the different options will be randomly selected from a few Japanese Hiragana syllables. HALFWAY THROUGH the task we will CHANGE THE AVAILABLE THREE OPTIONS which also give DIFFERENT AMOUNTS OF POINTS. The task will work exactly the same, also for the new three options! Before that happens we will notify you and also give you some time for another break. Please press F or J on

your keyboard to continue.
You finished the training! You can now get started with the task! The options A, B, and C are now represented by DIFFERENT SYMBOLS but everything works exactly as we showed you during the training. If you are still not quite sure what to do you can REPEAT THIS TUTORIAL by pressing F on your keyboard. If you want to CONTINUE TO THE TASK, please press J on your keyboard. Have fun and thank you for your participation! Press F to REPEAT this tutorial. Press J to CONTINUE with the task."

R1C08: The authors could do a better job at clarifying how de-correlated these surprise and uncertainty are in the "Surprise" model, which is like the "Uncertainty" model with a learning rate of 1.

The trial-by-trial correlation between surprise (absolute PE) and uncertainty was on average 0.502 for the Uncertainty model. The correlation between the absolute PE and uncertainty (*U*) depends on the π parameter (see Eq. 3 in the updated manuscript, $U_{k,t} = (1-\pi)U_{k,t-1} + \pi|PE_t|$). In principle, if π reaches 1 this means the uncertainty associated with a bandit is set to the last experienced absolute PE. Importantly, however, in our models uncertainty and surprise influenced behavior in different ways, which led the models to ultimately make different predictions. As you can see from Figure R2 above, the Surprise model did not appear to be confused very often with the Uncertainty model, or vice versa. We also note that even in the case of a learning rate of 1 the two models are not the same, in essence because the Surprise model adapts the learning rate immediately, i.e. on the same trial a large PE was experienced, while in the Uncertainty model any changes only come to influence the learning rate on the next trial.

Minor Comments

R1C09: The rationale for "free" and "guided" choices in the behavioral paradigm's explanation is not clear.

Thank you for bringing this to our attention. The guided choice trials were added so all bandits were sampled regularly. This was to avoid choice patterns in which participants never choose a specific bandit based on just one particularly low outcome. We added the following text to the methods section of the manuscript in which we clearly state the reason for adding guided choice trials and provide information on the consequences of not following the guided choice.

Ll. 570-575:

"To make sure all bandits were sampled regularly, the remaining trials consisted of guided choice trials (48 of 240 trials per run). In these trials, participants were instructed

to choose the bandit that was marked with a frame. Choices of the unmarked bandit resulted in no points and a reminder to choose the framed option without displaying the bandit's outcome before participants moved on to the next trial. All other task aspects were kept the same and correct choices awarded points as usual."

R1C10: Including the equation for the LME models implemented would improve readability.

We are thankful for the reviewer's engagement towards improving the manuscript's readability and happily incorporate the reviewer's suggestion. We now include the equations for the LME models in the methods section of the manuscript, together with short explanations. In places in which only the predicted variable differed but the model equation was otherwise identical we used references to previous model equations, also with the goal to improve readability. The exact changes we made are shown below.

Ll. 591-601:

"Behavioral analyses were done using linear mixed effects (LME) models with fixed effects of interest, such as bandit comparison (which bandits were presented to choose from, 3 levels, low-mid, mid-high, low-high), run number (2 levels), and age group (2 levels: older vs. younger adults). Models also included a random effect (intercept) of participant. The first of these models investigated overall performance (percentage of correct free choice trials) taking the form of

$$\begin{aligned}\mathrm{Performance_{free}} = \beta_0 + \gamma_{0,k} + \beta_1\,\mathrm{Group_{Age}} + \beta_2\,\mathrm{Run} + \beta_3\,\mathrm{Comp_{Bandit}} + \\ \beta_4\,\mathrm{Group_{Age}} * \mathrm{Run} + \beta_5\,\mathrm{Group_{Age}} * \mathrm{Comp_{Bandit}} + \\ \beta_6\,\mathrm{Run} * \mathrm{Comp_{Bandit}} + \beta_7\,\mathrm{Group_{Age}} * \mathrm{Run} * \mathrm{Comp_{Bandit}}\,,\end{aligned} \tag{5}$$

where β_0 and $\gamma_{0,k}$ denote global and subject-specific intercepts, β_1 to β_3 represent the fixed main effects of age group, run number, and bandit comparison, and β_4 to β_7 their respective interactions. A similar model was used to investigate choice speed (reaction times; all reaction times were collected in milliseconds and log-transformed before entering any analyses, equation identical to right hand side of Eq. 5)."

Ll. 605-610:

"This was compared to choices in low-mid trials following less surprising outcomes from the 20th to 40th percentile of the distribution. The model for this analysis was specified as

$$p(\text{Choice}_{\text{mid}}|\text{Trial}_{\text{low-mid}}) = \beta_0 + \gamma_{0,k} + \beta_1 \text{Position} + \beta_2 \text{Group}_{\text{Age}} + \beta_3 \text{Run} + \beta_4 \text{Position} * \text{Group}_{\text{Age}} + \beta_5 \text{Position} * \text{Run} + \beta_6 \text{Group}_{\text{Age}} * \text{Run} + \beta_7 \text{Position} * \text{Group}_{\text{Age}} * \text{Run} \quad (6)$$

and included a fixed effect of position relative to the large surprise, i.e. absolute PE (pre vs. post, β_1), in addition to the fixed effects of age group (β_2) and run (β_3), their interaction terms (β_4 to β_7) as well as a global and participant-specific intercept (β_0 and $\gamma_{0,k}$, respectively).”

Ll. 630-634:

“This measure of distortion was analyzed using a LME model specified as

$$\text{Distortion}_{\text{Estimate}} = \beta_0 + \gamma_{0,k} + \beta_1 \text{Group}_{\text{Age}} + \beta_2 \text{Run} + \beta_3 \text{Option} + \beta_4 \text{Group}_{\text{Age}} * \text{Run} + \beta_5 \text{Group}_{\text{Age}} * \text{Option} + \beta_6 \text{Run} * \text{Option} + \beta_7 \text{Group}_{\text{Age}} * \text{Run} * \text{Option} \quad (7)$$

with fixed effects of age group (β_1), run (β_2), and available options (low-mid vs. mid-high, β_3) as well as the respective interaction terms (β_4 to β_7) and a global and participant-specific intercept (β_0 and $\gamma_{0,k}$, respectively).”

Ll. 727-730:

“To quantify how well the model captures individual decision processes and to assess its plausibility, we repeated the above reported analysis of free choices in low-mid trials before and after the model encountered a surprising outcome of the mid bandit (see Eq. 6), but on the artificial data.”

R1C11: Including some model equations in the illustrative Fig. 2 would also help with readability and working memory demands for interpreting betas

We have now added the model equations to the figure. An updated version of the Figure has been added to the manuscript and has been reproduced below.

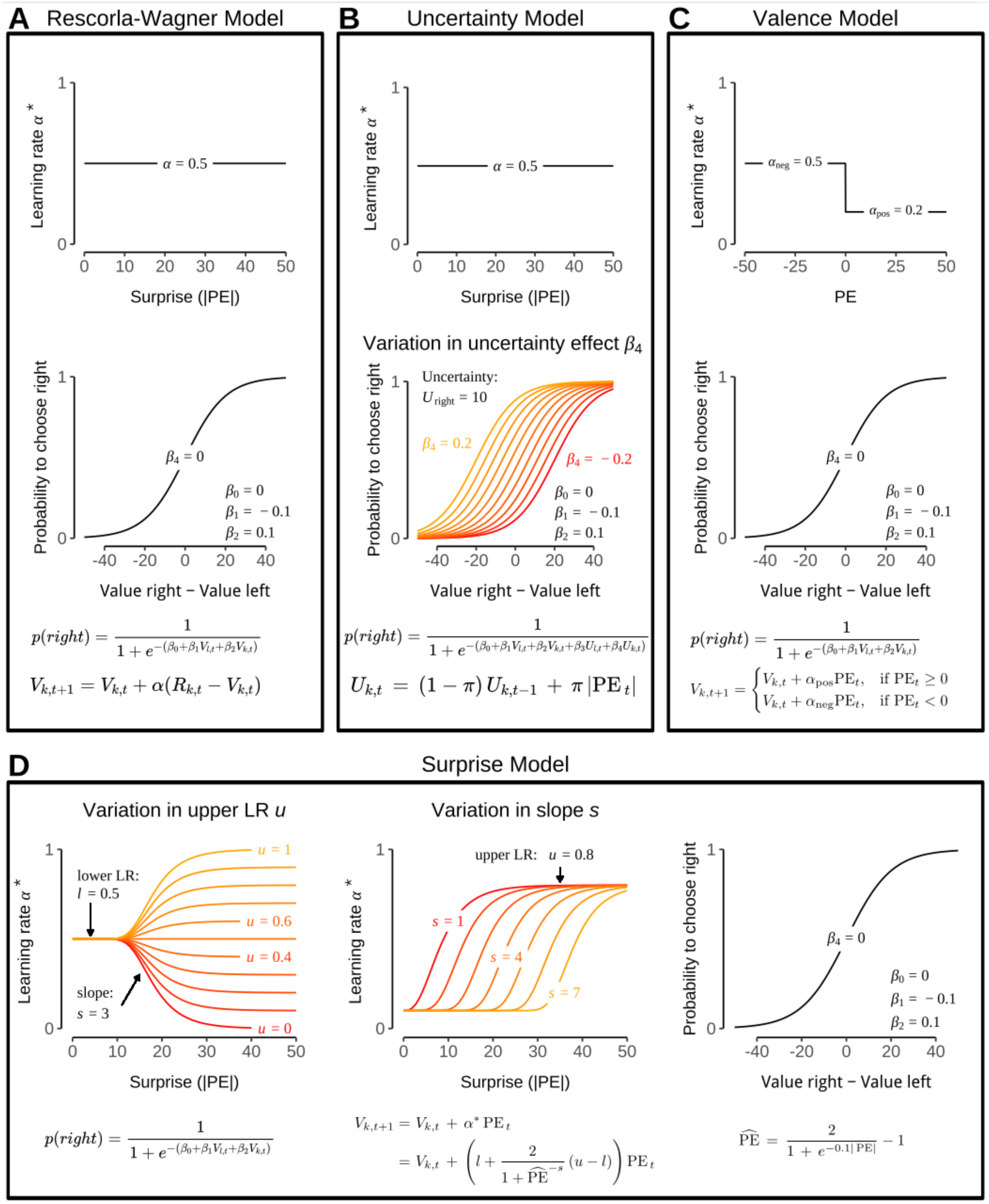

R1C12: Figure 2: k/l notation is confusing, why not say Left and Right?

We thank the reviewer for bringing this to our attention. We agree that the k/l notation added unnecessary complexity to the figure and updated the respective plots to use left/right instead. You can find the updated figure in the response to the previous point (R1C11).

R1C13: Line 252 – suggesting age-related neural differences is abrupt.

We have removed the mention of this literature. The new paragraph states (ll. 661-666):

> "*Previous studies have presented compelling evidence for differences in the way humans learn from positive and negative feedback (...) such as positivity or negativity biases (...). Older age has been shown to influence this difference (Frank & Kong, 2008; Eppinger & Kray, 2011).* "

R1C14: AIC was used for model fitting – wouldn't BIC be a better choice when comparing models with variable numbers of free parameters as this metric has a penalty for # of parameters.

Thank you for this suggestion. We first would like to clarify that we used the corrected Akaike information criterion (AICc, Sugiura, 1978; Cavanaugh, 1997) which does include a penalty for the number of free parameters. This penalty is less severe than the one used in the BIC, but more severe than the one used in the standard AIC. The AICc penalty incorporates the consideration of sample size and was developed to combine aspects of the AIC and BIC and to thereby overcome known issues of over- and underfitting using these metrics (Burnham & Anderson, 2002; Burnham & Anderson, 2004). Hence, our results do already include a penalty for the number of free parameters. We also note that a widely cited paper on comparing BIC and AIC in the context of psychology has described the choice as follows (Vrieze, 2012, Psychological Methods): "The choice between the AIC and BIC depends on one's notion of the true model. If the true model is assumed to be complex, with large, moderate, and small effects, and the candidate models oversimplifications, then the AIC may be preferred to the BIC. (...). To betray our bias, we expect the true model is quite complex in many areas of psychology". In our paper, it seems very likely that the true model is not in the candidate set and our best fitting model is only a good oversimplification. Hence, AICc seems a preferable choice.

We nevertheless followed up on the reviewer's suggestion and implemented a full model recovery process based on the BIC. This showed that the BIC offers decreased model recovery rates in the majority of the investigated models, while showing a strong tendency to underfit and falsely recover models with few parameters. Because of these results we argue in favor of not

changing the AICc-based model comparisons in the manuscript. Results of model recovery are shown below in Figure R3.

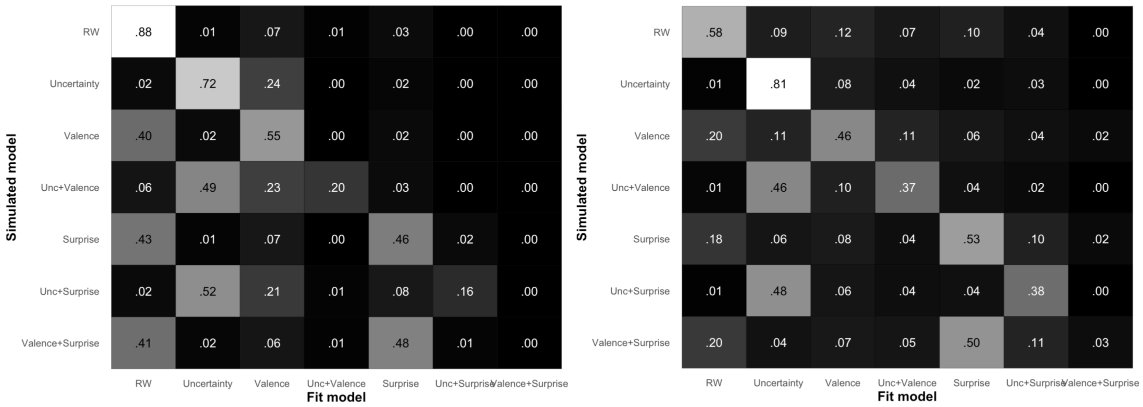

Figure R3. BIC-based model recovery (left) and AICc-based model recovery (right).

While the model recovery results using both criterions show several similarities, BIC-based model selection leads to a substantially increased chance to falsely recover the RW model when the true model was the Valence (40% vs. 20%), Surprise (43% vs. 18%), or Valence+Surprise model (41% vs. 20%). The correct recovery of models with higher numbers of parameters is lower when using BIC (e.g. in case of Unc+Valence 20% using BIC vs. 37% using AICc, or in the case of Unc+Surprise models 16% vs. 38%). On the other hand, the correct recovery of the RW model works better with BIC (88% using BIC vs. 58% using AICc).
In sum, this pattern is consistent with the literature mentioned above that suggests a tendency of the BIC to underfit. Since the rates of correct model recovery in our scenario generally decrease when using BIC (with the exception of the RW and Valence model) and it seems to add a bias towards more simple models we would argue against switching the information criterion used in the manuscript to BIC.

R1C15: Figure 3c and e – difficult to tell which group-level comparisons are significant.

Thank you for bringing this to our attention. We don’t show group level significance indicators in panel C/E since Fig. 3D (and F, for the RT data) already specifically highlight the results of the group-level comparison. In this context, we think that adding significance indicators to panels C (and E, for RT data) would overburden the plot with limited added value.

R1C16: Figure 3d – two older adults who displayed higher error for mid-high versus low-mid are unexpected. Does behavior correlate with estimate accuracy? How accurately do these participants estimate the likely outcome of respective choices? Are there individual differences in learning rates fit using the valence model related to performance (i.e., for these individuals I'd expect increased sensitivity to negative outcomes (higher α negative) which may explain the performance differences).

We thank the reviewer for these interesting questions and the opportunity to provide further detail. First, when specifically focusing on the two older outlier participants in Figure 3D and looking at their estimation performance, the data of both participants was flagged for exclusion by the criteria specified in the main manuscript (ll. 512-520). For one participant both runs were excluded, for the other only the second run was excluded. This remaining run, however, also showed low accuracy in the outcome estimation of all three bandits. This is shown in Figure R4 below, with the remaining run of the outlier participant marked in yellow.

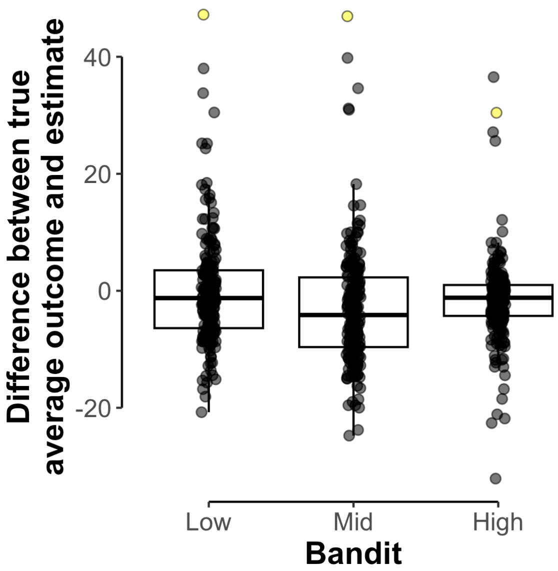

Figure R4. Mean difference between true average outcome of bandit (running average) and participants' estimates for all three bandits. Values above 0 indicate overestimation. Outlier participant shown as yellow dot.

Nonetheless, the relationship between estimation accuracy and behavior is also informative for the remaining sample. Here we specifically focus on the behavioral effect mentioned in the comment, if participants made relatively more errors in low-mid trials compared to mid-high trials (as shown in figure 3d in the main manuscript). Since this behavioral effect is averaged over runs, we only included participants that had valid choice and estimation behavior in both runs (n = 91, 44 younger, 47 older adults). The behavioral effect was significantly correlated with estimation accuracy in the low bandit ($r = .21$, $t(89) = 2.01$, $p = .047$) and the mid bandit ($r = -.23$, $t(89) = -2.21$, $p = .030$). Therefore, as expressed by the direction of the correlation

coefficients, stronger overestimation of the low bandit is related to relatively more errors in low-mid trials compared to mid-high trials. Similarly, a stronger underestimation of the mid bandit is related to the same effect. This is in line with the idea of the task, since non-optimal choices in low-mid trials should become more likely when the average outcomes of the low and mid bandit are perceived to be more similar.

As suggested by the reviewer, the behavioral effect is also correlated with model parameters. When focusing on the Valence model, more errors in low-mid trials are positively correlated with higher values of α_{neg} (r = .45, t(100) = 5.12, p < .001). Higher sensitivity for negative outcomes therefore is related to more often choosing the low bandit over the mid bandit in which individuals experience the largest negative prediction errors. Similarly, when focusing on the surprise model, the *u* parameter is positively correlated to the behavioral effect (r = .50, t(100) = 5.85, p < .001). Participants that show strong updating from high prediction errors (high *u*) also show more errors in low-mid trials compared to mid-high trials as the high prediction errors in the mid bandit are predominantly negative.

R1C17: The statement line 363 "the result cannot be explained by age group differences in risk aversion ..." was not clear

We thank the reviewer for pointing this out. We have now rephrased the sentence as follows (ll. 192-196):

> *"No evidence of a main difference between age groups in mid-bandit choices on average was found (X(1) = 1.363, p = .243). Given that risk aversion would lead participants to avoid the more variable mid bandit, it appears that risk aversion (or age differences therein) cannot explain the result reported above."*

R1C18: Line 393 – typo "heighted learning from to such singular events"

Thank you for spotting this. We corrected the typo and the sentence now correctly reads *"(...) heightened learning from such singular events"* (l. 222-223).

R1C19: For the next sentence, are the authors referring to trials in which outcomes fall in the second mode of the mid distribution, but below the mean?

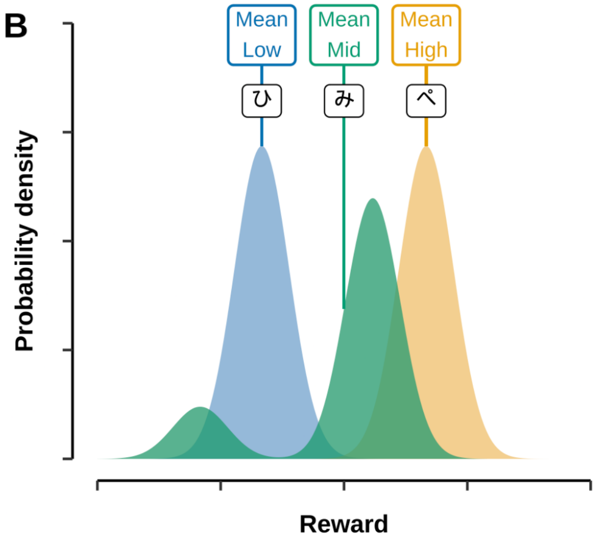

We thank the reviewer for bringing this issue to our attention. With the term *“low-mid”* we refer to trials in which the two arms of the bandit presented to the participant were the stimuli associated with the low and mid reward distributions. Based on Figure 1B in the manuscript (reproduced above), a “low-mid” trial would present ひ vs. み. A “low-mid” trial is therefore not defined by the outcome or if the outcome is below or above the mean of a distribution. It is only defined by the two distributions the outcome will be sampled from, given the participant’s choice for one of the bandits. The same is true for “mid-high” trials (み vs. ぺ, in this example) or also “low-high” trials (ひ vs. ぺ, in this example).
To make this terminology more evident to the reader we now introduce it more thoroughly when first mentioning pairwise bandit comparisons (ll. 132-135):

> *“This produced three trial types, which reflect the pair of bandits participants could choose from: low-mid trials featured bandits with low and medium means; low-high and mid-high trials offered the other respective bandit pairs.”*

R1C20: Line 334 - are authors referring to the outliers in the OA or YA group? This seemed a bit arbitrary.

We thank the reviewer for bringing this to our attention. In the sentence in question, we were specifically referring to the observations of two older adults, which was not apparent in the text. To avoid any misunderstandings, we now specify the data points we are referring to more directly by explicitly mentioning the age group and Cook’s distance suggesting them as influential data points.

Ll. 162-164:

”(...), this was likely caused by two relatively influential observations in the older age group (see Fig. 3D, Cook’s distance > 0.1). A direct comparison of the difference between low-mid vs. mid-high trials (...)”

Reviewer 2

Reviewer Summary

This paper combines clever experimental manipulation with state-of-the-art computational modeling to assess how younger and older adults differ in how they learn from surprising outcomes. The results indicate that choice behavior of most participants is best described by a model in which the learning rate depends on surprise, that is, the absolute size of prediction errors. Also, they show that, if anything, older adults’ choice behavior directly after surprising outcomes is affected more than that of younger adults.
The authors have to be applauded for their open science and good research practices: they openly shared their data and analysis code, considered a set of competing computational models, performed model and parameter recovery, reported analyses with and without outliers (instead of simply removing them), corrected *p*-values for multiple comparisons, did posterior predictive checks, and reported results that challenge their own hypotheses. The topic is timely and the proposed computational models help develop the reinforcement-learning field.
I do, however, have several concerns about the experimental design and computational models, and think the conclusions are formulated too strongly based on the results. I therefore believe the manuscript requires major revision (and likely additional experimental work). Below I explain why.

Major Comments

R2C01: Most importantly, although the bimodal distribution is a clever way to generate surprising outcomes, I wonder whether the experimental setup really allows one to disentangle the effects of surprising outcomes, mean differences and uncertainty. Specifically because the “mid” distribution has more surprising outcomes, a different mean outcome, and a larger uncertainty than the other two distributions. Although computational models should be capable of separating such explanations, their inability to reproduce behavioral results hint on that they are not in this particular situation. Why not just compare choices between a bimodal distribution and a unimodal distribution with the same mean outcome and standard deviation?

We thank the reviewer for this interesting suggestion, which we will discuss further below. Beforehand we would like to note that our core behavioral effect shown in Figure 4 does rely on within-bandit comparison, not a comparison between bandits. Specifically, in Fig 4A we depict only choices in which participants decided between low versus mid bandits. We compare the trial following a rare outcome with the trial before the surprising outcome and find a change in behavior. Hence, while the mid bandit per se has indeed a different mean and uncertainty than

other bandits, our argument does not rely on how participants behave towards the mid bandit relative to other bandits per se. Rather, choices in the same bandit comparison serve as a baseline for our effect. In the manuscript we also report that older and younger adults do not differ in their average percent choice of the mid bandit per se, yet they differ in their reaction after the critical surprise trials. We therefore believe our finding cannot be explained by bandit mean or uncertainty, but rather relates to individual trials characterized by large prediction errors, as described in the paper.

These considerations aside, we do agree that a setup with a bandit that has an identical mean and standard deviation could theoretically have benefits. But in a revised design we would still need the original three bandits, leading to 4 instead of 3 bandits. Given the constraints in how long older adults can perform such a task in a concentrated fashion, such a design would inevitably lead to cuts in the number of those trials that are most critical, i.e. trials in which the second mode of the mid bandit is experienced. We estimate that we would have about 25% fewer critical trials per participant in such a design (15 instead of 20), a reduction in power that we believe represents a major challenge.

An investigation of model recovery also indicates that our surprise model does produce unique patterns of behavior in our design. As can be seen in the Figure R5 below, the Surprise model is falsely recovered as one of the other 6 models in only 2-18% of cases, whereas it is correctly recovered in 53% (for comparison: the Rescorla Wagner model had a correct recovery rate of 58%). Although these numbers are not ideal, they do indicate with a large enough sample the models are identifiable. Note that model recovery does not indicate an inflationary recovery of the Surprise model when other models are true. We therefore believe this indicates that our design is suitable in principle. Finally, we want to note that the postdoc leading this project has now left academia. Hence, additional data acquisition is difficult to implement.

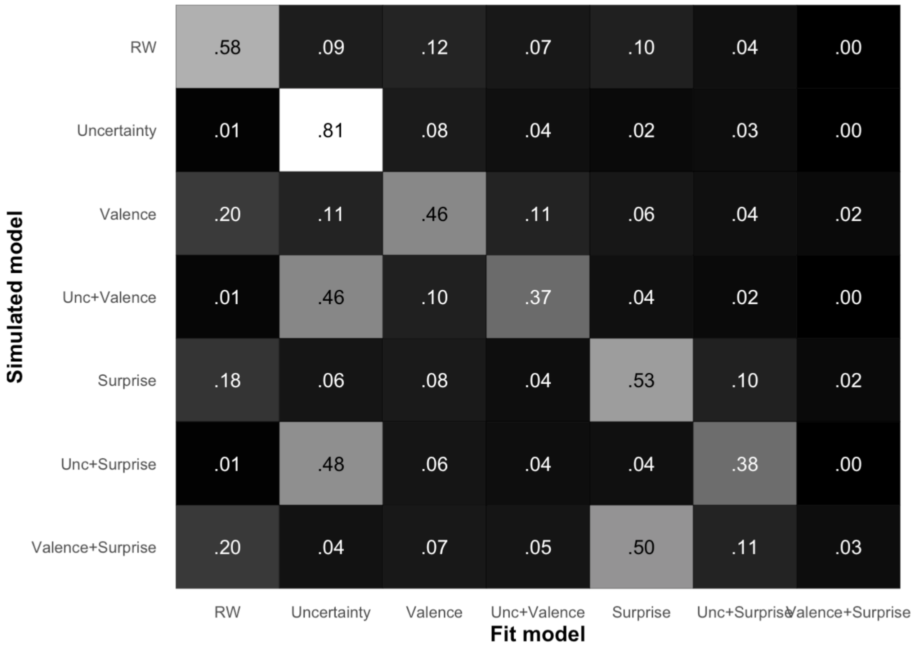

Figure R5. AICc-based model recovery.

R2C02: To better assess whether the previous point requires additional experimental work, it would be helpful to include predictive plots showing how the different models in your model set give rise to different patterns of choice behavior (see e.g., Collins & Frank, 2012, European Journal of Neuroscience). This would also clarify how valence biases translate to choice behavior in your task.

We thank the reviewer for this useful suggestion. We conducted simulations using the Surprise, Uncertainty, and Valence models with different sets of parameter values and created a set of predictive plots. To address the reviewer's comment, we focused on the immediate influence of surprising outcomes and how the different models are able to capture the drop in probability to choose the mid bandit in low-mid bandit comparisons after experiencing a surprising outcome evident in the behavioral analysis.

The simulated data shows that, given suitable parameters, the Surprise model and the Valence model can capture the central post surprise effect well, white the Uncertainty model can only reproduce a much more nuanced behavioral effect (Figure R6). Hence, the Surprise model *can* capture the observed behavior. Since the model predictions are richer than the behavior in these specific trial trial types, we have employed the model fitting described in the paper. Crucially, we also report model recovery results in our paper, which suggest that the core models are rarely confused with each other (see the matrix from our above answer). Specifically, when choices are simulated with the Surprise model the Valence model was wrongly recovered in only 8% of cases (6% vice versa) and the Uncertainty model was recovered wrongly in 6% of cases (2% vice versa). When the data were simulated by the Valence model, the Uncertainty model recovered them in 11% of cases (8% vice versa). We therefore suggest that the given task design using a bi-modal distribution offers an appropriate way to translate the individual characteristics of each model into different behavioral patterns that are distinguishable from each other.

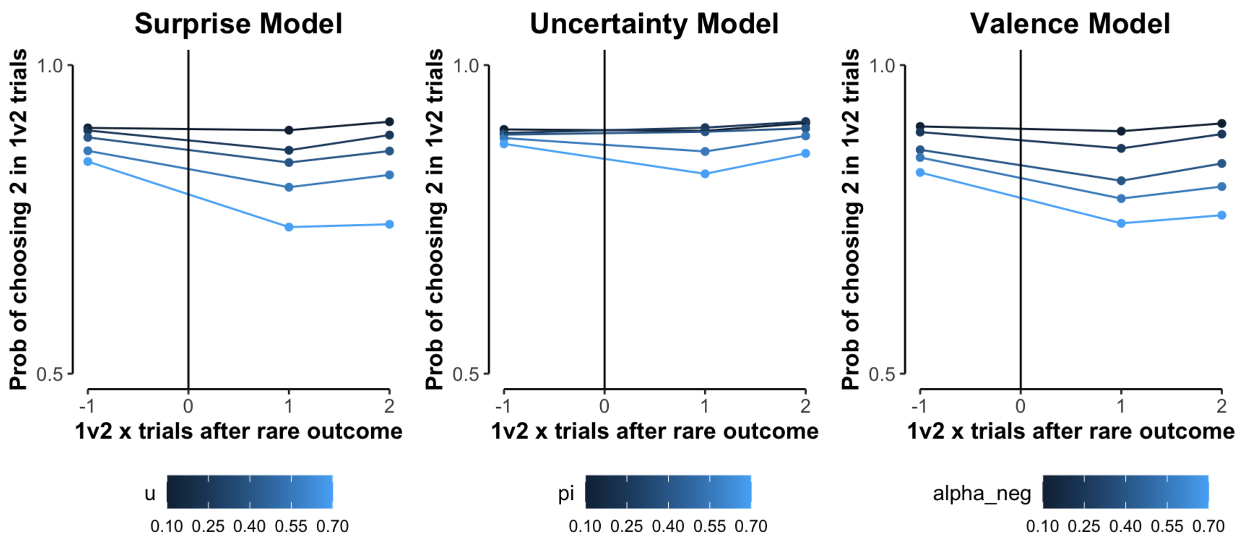

Figure R6. Simulations of choice patterns for Surprise, Uncertainty, and Valence models.

To simulate the data we used the following parameters:
Surprise model: l = 0.25 and s = 2.5, varying u
Uncertainty model: α = 0.25, varying π
Valence model: α_{pos} = 0.25, varying α_{neg}.

> **R2C03:** The authors present a very complete set of results, including results that challenge the authors' hypotheses, which has to be complimented. However, based on these results, the conclusions are formulated too strongly in favor of the proposed hypothesis. Looking at the individual modeling results in Figure 5, data from most participants are best described by a Surprise model. However, the data of the majority of participants are best described by one of the other models. This means there are large individual differences, not that surprise forms the best description of the results.

We agree with the reviewer. We have therefore updated the abstract, where we now state (ll. 13-15):

> *"Computational models indicated that learning rates in younger as well as older adults were influenced by surprise, rather than uncertainty, but also suggested large interindividual variability in the process underlying learning in our task."*

In addition, we also state this clearly in the discussion (ll. 379-383):

> *"Model comparison indicated that the Surprise model offered the best explanation of participants' decisions overall. Yet, a closer inspection also revealed that our data was characterized by large interindividual variability in the model that best explained different participants' data, and that fitted model's parameters did not reflect the age differences evident in the behavioral analyses."*

R2C04: The model-recovery results in Figure S3 warrant nuanced conclusions even further. A quick recalculation suggests that when the Surprise model fits the data best, this model was indeed the underlying model in only 63% of cases. This value is even worse for the other models, suggesting the considered computational models can describe the same pattern of choice behavior in the administered experiment.

To address the reviewer's concern, we have added this to the discussion as well (continuing right after the above statement (ll. 380-385):

> *"Yet, a closer inspection also revealed that our data was characterized by large interindividual variability in the model that best explained different participants' data, and that fitted model's parameters did not reflect the age differences evident in the behavioral analyses. We suspect that these findings are partly caused by issues of model identifiability, as evidenced by the reduced model recovery (see Fig S3)."*

R2C05: As the authors state themselves on line 396-398, you need the computational models to assess "whether this behavioral pattern can be explained best by surprise, or whether it might rather relate to uncertainty, differential learning from positive and negative prediction errors, or any combination of these factors." Conclusions about surprise should thus not be drawn based on behavioral results (as in section Effects of highly surprising events on subsequent choices).

We thank the reviewer for drawing our attention to the statement. In short, we did not mean to imply that the behavioral analysis is unable to dissociate the effects - for example, the effect of surprise and weighing of positive and negative outcomes can in fact be dissociated behaviourally. Rather, the change in choice following a surprising outcome is a key prediction of the surprise account, and was the core motivation of our study. We apologize if our previous wording was unclear and have adapted the text accordingly, see below.
The motivation for the modeling was that while this effect is indicative of surprise processing, one needs to be careful in assuming that this prediction is *exclusive* to the surprise account. The main benefit of modeling is that models constitute a precise formalization of hypothesis space and allow us to compute predictions not only for a certain key behavior, but for the entire sequence of choices as implied by the model's generative dynamics.

We recognize that the way we formulated the sentence is not clear on this point, and therefore re-formulated the sentence as follows (ll. 226-231):

”Building on these behavioral results, we used computational modeling to specifically contrast the contributions of surprise, uncertainty and differential learning from positive and negative prediction errors (as well as combination of these) to behavior. While the behavioral effect following surprise trials reported above is qualitatively consistent with our hypothesized mechanism, computational models allow us to test a more precise version of our hypothesis across the entire sequence of choices.”

We have also carefully reread the section “Effects of highly surprising events on subsequent choices,”. To the best of our ability we believe that we only report statistical models and statistical tests without offering any interpretation at this point.

R2C06: Assuming you can disentangle the considered processes using computational models, I believe it important to add a model including both valence and surprise. The current results indicate these two models fit the data best, suggesting both processes underlie choice behavior (potentially concurrently).

Both reviewers have suggested this change and we thank both of them for this contribution, which we are happy to implement. This combined model includes the key feature of the Surprise model, a mapping between learning rate and magnitude of prediction error (surprise), but also allows learning rates to be different for positive vs negative prediction errors.

Fitting this model to our data showed that it had an overall higher AICc than many of the other models, and that only a small number of subjects were fit best by it, as shown in Figure R1 below. In addition, we found that the combined Valence+Surprise model had poor model recovery: data simulated using the Valence+Surprise model was only correctly assigned to the Valence+Surprise model in 3% of the cases, which was notably lower than the recovery rate observed in all other models (see Figure R2 below). In most cases, the recovered model was the simpler Surprise model (50%) which does not account for prediction error valence.

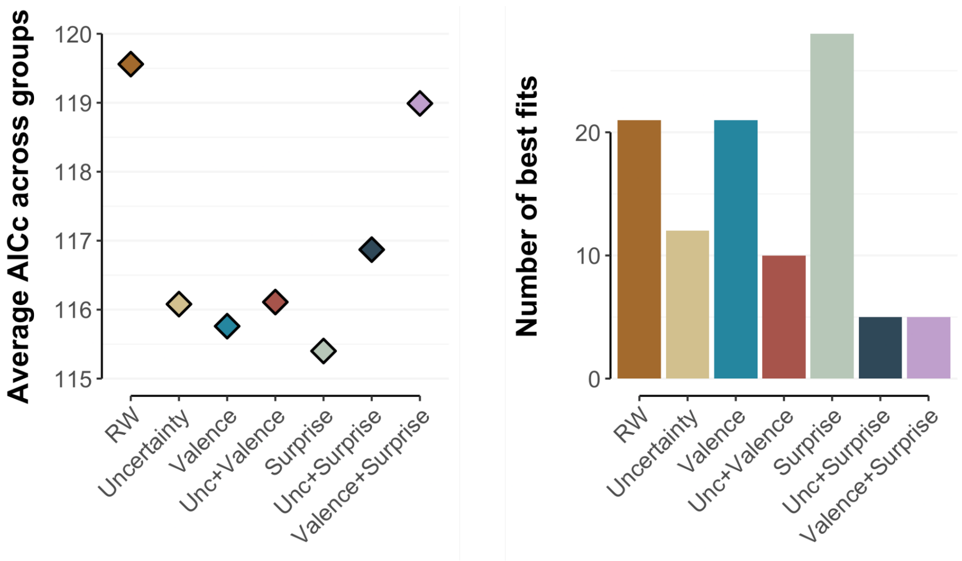

Figure R1. Left: Total average AICc values for the novel Valence+Surprise model in comparison to all other models. Right: number of participants best fit by the novel Valence+Surprise model in comparison to all other models.

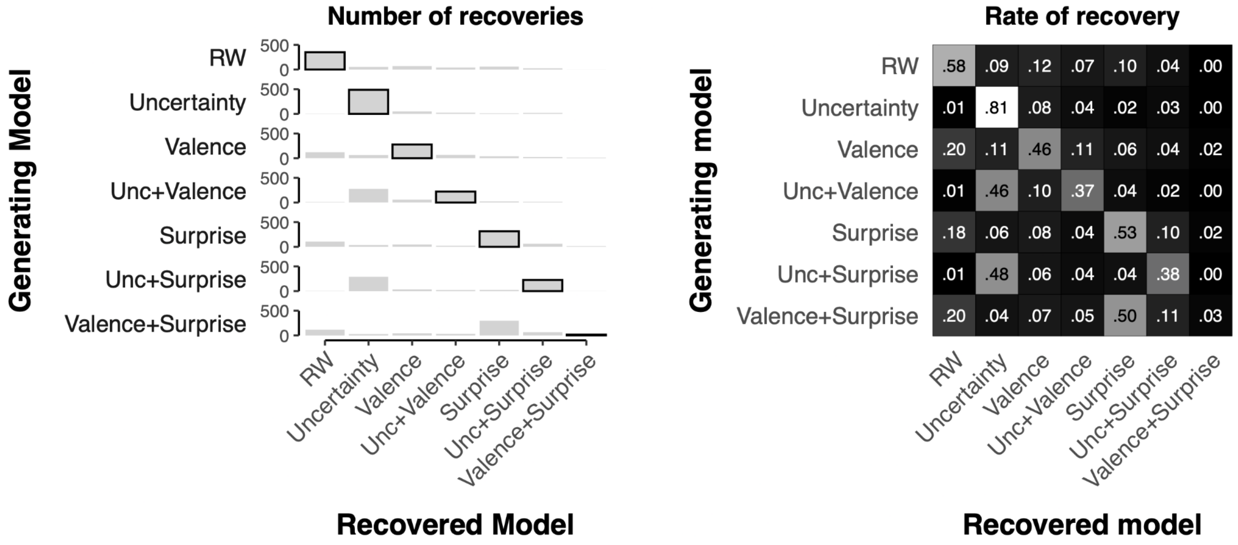

Figure R2. Model recovery including the novel Valence+Surprise model (bottom most model), in comparison to all other models. Left plot shows the absolute number of recoveries, the right plot shows relative rates of recovery. The combined model had notably poor recovery.

Given that the Surprise+Valence model did not provide a compelling account of behavior, in combination with the nature of our design that is not ideal to test such a model, we decided to report information about the new Surprise+Valence model mostly in the SI of the manuscript, while adding the following notes in the main text that inform the reader of the additional model and its results:

Ll. 695-699 (Methods):

“We also implemented an additional model that combined the functionality of the Surprise model with that of the Valence model potentially capturing if surprise from positive or negative prediction errors differently affects learning. This model did not provide any superior fits compared to the winning models reported here. Details can be found in the SI.”

Ll. 279-281 (Results):

“Results of an additional model that combined the functionality of the Surprise and Valence models can be found in the SI.”

R2C07: It is unclear to me whether the goal of the paper is to assess the effects of surprise on learning (as the title and the final introduction paragraph suggest) or to separate effects of surprise and uncertainty (as other introduction paragraphs suggest). I really lost track when, on lines 262-263, you stated that “our second main interest was to ask whether large absolute prediction errors, i.e. surprise, influenced learning rates”, making me wonder what your first main interest was.

We thank the reviewer for this comment which was very helpful for improving our communication of the study’s main goals. The main goal of the study was to assess the effects of surprise on learning in older relative to younger adults. Since uncertainty processing plays an important role in this context (e.g. interpretation of results, previous research in the aging field) it was vital to us to mention uncertainty and its relevance towards our goal early on. To avoid the impression that the separation of effects of surprise and uncertainty was the main goal rather than a necessary step we made some adjustments to the manuscript.
We now emphasize the main goal of the study by having a more clear formulation of the study’s approach and main question and a more straightforward statement about its hypotheses (ll. 57-95, statements about hypotheses marked in bold):

“Past aging research has studied related but not identical aspects of decision making (Nassar et al., 2016; Mata et al., 2011; Pachur, Mata, & Hertwig, 2017). Nassar et al. (2016), for instance, investigated learning of older and younger adults in changing environments characterized by so called non-stationary bandits, i.e. a scenario in which the rewards associated with different actions change over time. They specifically focused on how participants modulated their learning rates in response to outcome deviations that reflected a true shift of the bandit mean (due to an environmental change point) versus merely a random deviation due to variability around each bandit's mean, which represent a mix of outcome probability and deviation from previous events. Nassar et al. suggested that in this setup uncertainty processing, but not surprise processing, is impaired in older relative to younger adults Nassar et al., 2016). These effects might arise from a simplified learning strategy that reduces cognitive resource expenditure, making older adults less sensitive to smaller prediction errors that can be

attributed to uncertainty compared to larger and more surprising prediction errors (Bruckner, Nassar, Li, & Eppinger, 2020). However, this line of work leaves open the question of how surprise affects learning in older adults when the surprising event does not signal a fundamental change point and, therefore, dictates a lower learning rate (Nassar, Bruckner, & Frank, 2019).

Other work in the domain of decisions from description suggests that older adults overweight low probability events in the gain domain (i.e., show more risk-seeking behavior), compared to younger adults (Pachur et al., 2017). This work has focused purely on how the stated probability of events affects decision making. In contrast, when decisions are based on learned probabilities, referred to as decisions from experience (Hertwig & Erev, 2009; Wulff, Mergenthaler-Canseco, & Hertwig, 2018), age-related differences in choice behavior and risk-taking often differ compared to decisions from description, where age differences in risk preferences depend on the exact choice scenario (Mata et al., 2011).

Therefore, we aimed to examine age-related learning and decision making differences in an experience-based choice task with stationary outcome probabilities. We specifically studied the effects of outcomes that are highly surprising, i.e. differ significantly from most previous outcomes. ***We stipulated that surprise could affect the learning rate with which participants update their expectations in a trial and error setting, even when dissociated from the effects of probability. In line with previous work, we expected that surprise would have a greater effect on older adults, as compared to younger adults.*** *Taking a reinforcement learning (RL) perspective (Sutton & Barto, 2018; Dayan & Daw, 2008), we conceptualized surprise as the absolute prediction error (PE), i.e. the deviation of an observed outcome from the current expectation.* ***While standard RL theory assumes that prediction errors are weighted by a constant learning rate parameter α ∈ [0, 1], we hypothesized that learning rates are modulated by the absolute PE, i.e. surprise of a given trial. Our idea specifically predicts that surprise impacts learning immediately, i.e. affects the update on the very same trial that caused the surprise.****”*

Additionally, the paragraph in which we mainly mention the differentiation between surprise and uncertainty is now introduced in relation to the main goal (ll. 101-103):

“We designed a novel task in which participants learned from outcomes drawn from a stationary bimodal distribution (a non-changing distribution with two peaks) that yielded a number of benefits when studying the effects of surprise on learning.”

Finally, we changed the confusing wording in the methods section the reviewer cited in their comment (ll. 680-681):

“Surprise model. *This model asked whether observing surprising outcomes would influence participants' learning rate, compared to observing less surprising outcomes.”*

R2C08: The introduction would benefit from more theoretical discussion. I'm no expert in aging so reading the introduction, I wonder why aging would have an effect on learning from surprise. It may help to refer to studies on surprise in the imaging field, including work from one of the authors himself (Nassar et al., 2019, eLife).

Thanks for the suggestion to motivate our hypotheses about older adults more thoroughly. We have extended the introduction, and in line with the reviewer's suggestion, cited our previous work on learning under uncertainty focusing on surprise in younger adults (Nassar et al., 2019) and older adults (Bruckner, et al., 2020). Since the updated paragraph (ll. 57-81) was already cited in our answer immediately above, we will not repeat it here.

R2C09: I miss reference (in the introduction, discussion or both) to a large literature in the decisions from experience field on the influence of surprise. For example, by Erev and colleagues (Nevo & Erev, 2012, Frontiers in Psychology), who repeatedly showed underweighting of rare events in decisions from experience (e.g., Hertwig et al., 2004, Psychological Science) as opposed to the observed overweighting in the current study.

Thank you for this suggestion. We have addressed this point together with our response to comment R2C08.

R2C10: To not further delay the revision process, I will try whether the open data and code are easily accessible in the next revision round.

Thank you. We have posted the updated code and data on the two following repositories (please note that the DOIs from the original submissions now point to an older version).
Data: https://gin.g-node.org/koch_means_cook/pedlr-derivatives/src/plos_review_01
Code: https://github.com/koch-means-cook/pedlr/tree/master

Minor Comments

R2C11: Especially in the beginning of the paper you assume quite some prior knowledge. For example, to readers outside of the reinforcement-learning field "asymmetric outcome distributions, which decouple outcome magnitude, probability, uncertainty, and surprise" (lines 6-8) and "non-stationary bandits" (line 61) would be difficult to understand without further explanation. Also, how do concepts like "react to surprising events" (line 47) and "uncertainty-averse" (line 51) relate?

We thank the reviewer for this suggestion and for helping us to make our manuscript more accessible to a wider audience. In response, we adapted the respective sections to be more clear in their wording and to include additional explanations:

Ll. 6-7:

> *"(...) we studied choices while participants learned from bandits with either Gaussian or bi-modal outcome distributions, which decoupled outcome probability, uncertainty, and surprise."*

Ll. 58-61:

> *"Nassar et al. (2016), for instance, investigated learning of older and younger adults in changing environments characterized by so called non-stationary bandits, i.e. a scenario in which the rewards associated with different actions change over time."*

Ll. 101-103:

> *"We designed a novel task in which participants learned from outcomes drawn from a stationary bimodal distribution (a non-changing distribution with two peaks) that yielded a number of benefits when studying the effects of surprise on learning."*

Ll. 106-108:

> *"Second, a bimodal distribution has a second peak of outcomes that are far from the mean, but still relatively probable, which makes it possible to decouple an event's probability from its surprise."*

Additionally, we made some edits to the introduction which we mentioned already in the response to above comments. We hope these will help to make our concepts and their relationship more clear. For example (ll. 67-70):

> "*These effects might arise from a simplified learning strategy that reduces cognitive resource expenditure, making older adults less sensitive to smaller prediction errors that can be attributed to uncertainty compared to larger and more surprising prediction errors (Bruckner, Nassar, Li, & Eppinger, 2020).*"

R2C12: The authors explicitly define surprise on line 74 (“We conceptualized surprise as large absolute prediction errors”), which is very helpful. The difference between surprise and rare outcomes is unclear though. Repeatedly, the authors emphasize that surprise is different from rare outcome (e.g., “a very surprising, but not necessarily very rare, event” (line 80); “older adults do not exhibit heightened sensitivity to rare events per se, but rather to events that elicited particularly large prediction errors (i.e. surprise).” (lines 536-538)). However, they also state: “To investigate the effect of large prediction errors, we analyzed free choices in low-mid trials before and after participants encountered a rare outcome of the mid bandit” (lines 187-198), suggesting surprise is the same as observing a rare outcome.

We thank the reviewer for raising this important issue. First of all, we sincerely apologize for the confusion that was specifically caused by the last sentence the reviewer cited (ll. 187-198 in the manuscript at submission). The usage of the word “rare” was a mistake we overlooked prior to submitting the manuscript and has now been edited to correctly say “surprising” (ll. 601-604):

> *“To investigate the effect of large prediction errors, we analyzed free choices in low-mid trials before and after participants encountered a surprising outcome of the mid bandit's lower mode (below the distributions 20th percentile, on average n = 4.71 and n = 4.44 choices per run/participant, respectively).”*

We also agree that the difference between rarity and surprise of an event has not become fully clear in the manuscript and have now made changes throughout the text to solve this. First, in order to make it more apparent to the reader *why* this distinction is important we now emphasize the fact that past research on the weighting of events has focused mostly on effects of probability and how *rare* events are, while our reinforcement learning approach focuses on the surprise elicited by an event:

Ll. 74-85:

> *“Other work in the domain of decisions from description suggests that older adults overweight low probability events in the gain domain (i.e., show more risk-seeking behavior), compared to younger adults (Pachur et al., 2017). This work has focused purely on how the stated probability of events affects decision making. In contrast, when decisions are based on learned probabilities, referred to as decisions from experience (Hertwig & Erev, 2009; Wulff, Mergenthaler-Canseco, & Hertwig, 2018), age-related differences in choice behavior and risk-taking often differ compared to decisions from description, where age differences in risk preferences depend on the exact choice scenario (Mata et al., 2011).*
> *Therefore, we aimed to examine age-related learning and decision making differences in an experience-based choice task with stationary outcome probabilities. We specifically studied the effects of outcomes that are highly surprising, i.e. differ significantly from most previous outcomes.”*

Ll. 95-100:

> *"In turn, this is akin to a process that, gives more weight to an event not based on its probability, but on its associated surprise which dissociates our proposal from previous work where learning rates only ramp up future, but not current, learning (Li, Schiller, Schoenbaum, Phelps, & Daw, 2011), and prediction error magnitude is often confounded with outcome probability (Pearce & Hall, 1980; Li et al., 2011; Jepma et al., 2016; O'Reilly, 2013; Nassar, Wilson, Heasly, & Gold, 2010)."*

Additionally, we now further highlight how our task, together with our approach, was specifically designed to separate an event's rarity from its surprise (ll. 101-112):

> *"We designed a novel task in which participants learned from outcomes drawn from a stationary bimodal distribution (a non-changing distribution with two peaks) that yielded a number of benefits when studying the effects of surprise on learning.*
> *(...)*
> *Second, a bimodal distribution has a second peak of outcomes that are far from the mean, but still relatively probable, which makes it possible to decouple an event's probability from its surprise. In unimodal Gaussian distributions, prediction errors, outcome probabilities, and magnitude are correlated. However, this correlation is lessened or absent in long-tailed or bimodal distributions, where outcomes with a relatively small difference from the mean can have a probability as low as outcomes much further from the mean."*

With these edits we hope that the differences between rare and surprising events and their role in distinguishing our study and approach from previous research have become more clear.

R2C13: To improve readability, I would move all behavioral results that do not directly answer research questions but serve as (sanity) checks to the supplements.

Improving the readability of the article in the spirit of making it less dense is a useful suggestion. To achieve this aim, we have moved the sanity checks of the Rescorla Wagner model to the Methods, and only mention these briefly in the main text (ll. 284-293):

> *"Basic analyses indicated that the RW account of value learning captured core aspects of participant behavior (see Methods). Yet, the RW model did not offer the best fit to participants' choices. Instead, the model comparison showed that the corrected AIC score (AICc, Cavanaugh, 1997) was lowest for the Surprise model (AICc: 115.40 vs. 119.56 of the RW model). The next best models were the Valence (115.76), Uncertainty (116.08), Unc+Valence (116.11), and Unc+Surprise (116.86) models (see Fig. 5A, AICc of RW model: 119.56). A protected exceedance probability analysis showed the Surprise model as the most likely data-generating process across all participants (77.0 %; via R package bmsR, [...]), followed by the Valence model (22.7 %, see Fig. 5B)."*

We also moved the detailed Methods to the end of the article, and provide a much briefer summary of the main information at the beginning of the results section (ll. 124-145):

"A total of 51 younger (18-30 years, avg.: 24.4) and 51 older (50-73 years, avg.: 57.2) participants performed a value-based choice task online. The task consisted of two runs á 240 trials in which participants were asked to learn about the value of three bandits, each indicated by a different Hiragana symbol (Fig. 1A). Outcomes ranged between 0 and 100 and the averages of the three bandits were set such that one bandit had a low, one a medium and one a high mean, each differing by 16.6 points on average from its neighbor (Fig. 1B). Participants learned about the average outcomes through free choice trials in which they could select one out of two offered Hiragana symbols and received an outcome sampled from the corresponding bandit's distribution (192 trials/run, Fig. 1A). This produced three trial types, which reflect the pair of bandits participants could choose from: low-mid trials featured bandits with low and medium means; low-high and mid-high trials offered the other respective bandit pairs.
Bandits not only differed in their mean, but also in their distribution. While the low and high bandits had symmetrical Gaussian outcome distributions (SD=5.55 points), the mid bandit had a bi-modal distribution with a main mode that generated 80% of outcomes and smaller mode that generated 20% of outcomes (Fig. 1B). This crucial manipulation allowed us to investigate how sensitive learning was to outcomes that had a large deviation from previously experienced outcomes, but were not the most rare outcomes. To provide enough experience with each bandit's outcome, we asked participants on 20% of trials to select a computer-determined bandit instead of choosing freely (forced choice trials). We also asked participants to directly provide an estimate of each bandit's value using a slider (16 trials / run, Fig. 1A, bottom)."

We have also shortened the model descriptions and moved some information to the Methods. These changes have made the Results section overall less dense and greatly improved the manuscript. We thank the reviewer for this useful suggestion.

R2C14: This could be a personal preference, but I think the conclusion paragraph would benefit from a one-sentence conclusion on whether "older adults show greater sensitivity to outcomes that elicit large absolute prediction errors compared to younger adults".

We agree that a clear summarizing sentence would be beneficial. We have now rewritten the summarizing paragraph to include such a clear conclusion. The relevant section now reads as follows (ll. 366-376):

"We found that behavioral accuracy in low-mid bandit choices was significantly lower compared to mid-high trials despite the fact that both bandit pairs exhibited the same difference in their mean outcome. This was particularly the case for older compared to younger adults. This suggests that surprising outcomes are overweighted, relative to ordinary outcomes, and that this effect becomes more prominent with age. This effect was also present in explicit value ratings, in which both age groups underestimated the difference in average rewards of the low and mid bandit, and older adults showed a

stronger tendency to do so. An analysis of detailed choice time courses also found that surprising outcomes had a stronger influence on consecutive choices in older adults compared to younger adults, suggesting a greater sensitivity to surprising outcomes in older adults."

R2C15: If I'm correct trial indices (i.e., t subscripts) are missing in equations 1, 5, 6, and 7.

We thank the reviewer for making us aware of the missing indices and apologize for the mistake. In response, we added the missing *t* subscripts to the prediction error terms in equations 1 and 5 (now Eq. 2, after our edits) where they were not correctly specified. This comment also helped us to understand that we were not concise enough in our description of the $\widehat{PE}$ term in equations 6 and 7 (now Eqs. 4, 10, and 11). This term is a constant that functions as a scaling factor in the definition of the relationship between learning rate and absolute prediction error. It therefore does not need a *t* subscript as it is independent of the trial at hand. We addressed this issue in the response to R2C16 below, which is specifically about this topic.

R2C16: It is unclear to me why you would use a transformation of the absolute prediction error instead of the error itself and how this term was derived.

In the classical RW model, *α* lies in the range between 0 and 1, and the scale of the prediction errors reflects the scale of the rewards (i.e. if rewards range between 0 and 100, the PEs will fluctuate (at most) in this range; but if rewards range from 0 to 1, the PEs will fluctuate in this range). Our model has two PE terms: the normal PE term, and the "PE hat" term, which the reviewer refers to. Unlike the normal PE, PE hat is a factor that scales the learning rate, which by definition should only lie in the range of 0 to 1. Hence, PE hat has to lie in the range of 0 to 1, regardless of the scale of rewards, whereby a value of 1 reflects maximally large PE. To achieve the rescaling of the absolute PE is the aim of the transformation the reviewer refers to.

We have now clarified this by adding the following statement to the manuscript (ll. 268-269):

"*The introduction of $\widehat{PE}$ in the equation of α^* was necessary to achieve rescaling into the range of [0, 1], which is needed for learning rates.*"

References

Bruckner, Rasmus, Matthew R Nassar, Shu-Chen Li, and Ben Eppinger. 2020. “Differences in Learning Across the Lifespan Emerge via Resource-Rational Computations.” *PsyArXiv*. https://doi.org/10.31234/OSF.IO/NH9BQ.

Burnham, Kenneth P., and David R. Anderson. 2002. *Model Selection and Multimodel Inference*. Edited by Kenneth P. Burnham and David R. Anderson. 2nd ed. New York, NY: Springer New York. https://doi.org/10.1007/b97636.

Burnham, Kenneth P., and David R. Anderson. 2004. “Multimodel Inference: Understanding AIC and BIC in Model Selection.” *Sociological Methods & Research* 33 (2): 261–304. https://doi.org/10.1177/0049124104268644.

Cavanaugh, Joseph E. 1997. “Unifying the Derivations for the Akaike and Corrected Akaike Information Criteria.” *Statistics & Probability Letters* 33 (2): 201–8. https://doi.org/10.1016/S0167-7152(96)00128-9.

Nassar, Matthew R., Rasmus Bruckner, and Michael J. Frank. 2019. “Statistical Context Dictates the Relationship between Feedback-Related EEG Signals and Learning.” *ELife* 8 (August). https://doi.org/10.7554/eLife.46975.

Sugiura, Nariaki. 1978. “Further Analysis of the Data by Akaike’s Information Criterion and the Finite Corrections.” *Communications in Statistics - Theory and Methods* 7 (1): 13–26. https://doi.org/10.1080/03610927808827599.

Vrieze, Scott I. 2012. “Model Selection and Psychological Theory: A Discussion of the Differences between the Akaike Information Criterion (AIC) and the Bayesian Information Criterion (BIC).” *Psychological Methods* 17 (2): 228–43. https://doi.org/10.1037/a0027127.
