# Supplementary material for: Influence of surprise on reinforcement learning in younger and older adults
Source: PLoS Comput Biol. 2024 Aug 14;20(8):e1012331. doi: 10.1371/journal.pcbi.1012331 (PMC11346965; doi:10.1371/journal.pcbi.1012331)
Supplement: Attachment — Submitted filename: surprise_review.docx [file pcbi.1012331.s002.docx]

Review of “Influence of surprise on reinforcement learning in younger and older adults”

This paper combines clever experimental manipulation with state-of-the-art computational modeling to assess how younger and older adults differ in how they learn from surprising outcomes. The results indicate that choice behavior of most participants is best described by a model in which the learning rate depends on surprise, that is, the absolute size of prediction errors. Also, they show that, if anything, older adults’ choice behavior directly after surprising outcomes is affected more than that of younger adults.

The authors have to be applauded for their open science and good research practices: they openly shared their data and analysis code, considered a set of competing computational models, performed model and parameter recovery, reported analyses with and without outliers (instead of simply removing them), corrected *p*-values for multiple comparisons, did posterior predictive checks, and reported results that challenge their own hypotheses. The topic is timely and the proposed computational models help develop the reinforcement-learning field.

I do, however, have several concerns about the experimental design and computational models, and think the conclusions are formulated too strongly based on the results. I therefore believe the manuscript requires major revision (and likely additional experimental work). Below I explain why.

Major points:

1. Most importantly, although the bimodal distribution is a clever way to generate surprising outcomes, I wonder whether the experimental setup really allows one to disentangle the effects of surprising outcomes, mean differences and uncertainty. Specifically because the “mid” distribution has more surprising outcomes, a different mean outcome, and a larger uncertainty than the other two distributions. Although computational models should be capable of separating such explanations, their inability to reproduce behavioral results hint on that they are not in this particular situation. Why not just compare choices between a bimodal distribution and a unimodal distribution with the same mean outcome and standard deviation?
2. To better assess whether the previous point requires additional experimental work, it would be helpful to include predictive plots showing how the different models in your model set give rise to different patterns of choice behavior (see e.g., Collins & Frank, 2012, *European Journal of Neuroscience*). This would also clarify how valence biases translate to choice behavior in your task.
3. The authors present a very complete set of results, including results that challenge the authors’ hypotheses, which has to be complimented. However, based on these results, the conclusions are formulated too strongly in favor of the proposed hypothesis.
   a. Looking at the individual modeling results in Figure 5, data from most participants are best described by a *Surprise* model. However, the data of the majority of participants are best described by one of the other models. This means there are large individual differences, not that surprise forms the best description of the results.
   b. The model-recovery results in Figure S3 warrant nuanced conclusions even further. A quick recalculation suggests that when the *Surprise* model fits the data best, this model was indeed the underlying model in only 63% of cases. This value is even worse for the other models, suggesting the considered computational models can describe the same pattern of choice behavior in the administered experiment.
   c. As the authors state themselves on line 396-398, you need the computational models to assess “whether this behavioral pattern can be explained best by surprise, or whether it might rather relate to uncertainty, differential learning from positive and negative prediction errors, or any combination of these factors.” Conclusions about surprise should thus not be drawn based on behavioral results (as in section Effects of highly surprising events on subsequent choices).
4. Assuming you can disentangle the considered processes using computational models, I believe it important to add a model including both valence and surprise. The current results indicate these two models fit the data best, suggesting both processes underlie choice behavior (potentially concurrently).
5. It is unclear to me whether the goal of the paper is to assess the effects of surprise on learning (as the title and the final introduction paragraph suggest) or to separate effects of surprise and uncertainty (as other introduction paragraphs suggest). I really lost track when, on lines 262-263, you stated that “our *second* main interest was to ask whether large absolute prediction errors, i.e. surprise, influenced learning rates”, making me wonder what your first main interest was.
6. The introduction would benefit from more theoretical discussion. I’m no expert in aging so reading the introduction, I wonder why aging would have an effect on learning from surprise. It may help to refer to studies on surprise in the imaging field, including work from one of the authors himself (Nassar et al., 2019, *eLife*).
7. I miss reference (in the introduction, discussion or both) to a large literature in the decisions from experience field on the influence of surprise. For example, by Erev and colleagues (Nevo & Erev, 2012, *Frontiers in Psychology*), who repeatedly showed *under*weighting of rare events in decisions from experience (e.g., Hertwig et al., 2004, *Psychological Science*) as opposed to the observed *over*weighting in the current study.
8. To not further delay the revision process, I will try whether the open data and code are easily accessible in the next revision round.

Minor points:

9. Especially in the beginning of the paper you assume quite some prior knowledge. For example, to readers outside of the reinforcement-learning field “asymmetric outcome distributions, which decouple outcome magnitude, probability, uncertainty, and surprise” (lines 6-8) and “non-stationary bandits” (line 61) would be difficult to understand without further explanation. Also, how do concepts like “react to surprising events” (line 47) and “uncertainty-averse” (line 51) relate?
10. The authors explicitly define surprise on line 74 (“We conceptualized surprise as large absolute prediction errors”), which is very helpful. The difference between surprise and rare outcomes is unclear though. Repeatedly, the authors emphasize that surprise is different from rare outcome (e.g., “a very surprising, but not necessarily very rare, event” (line 80); “older adults do not exhibit heightened sensitivity to rare events per se, but rather to events that elicited particularly large prediction errors (i.e. surprise).” (lines 536-538)). However, they also state: “To investigate the effect of large prediction errors, we analyzed free choices in low-mid trials before and after participants encountered a rare outcome of the mid bandit” (lines 187-198), suggesting surprise is the same as observing a rare outcome.
11. To improve readability, I would move all behavioral results that do not directly answer research questions but serve as (sanity) checks to the supplements.
12. This could be a personal preference, but I think the conclusion paragraph would benefit from a one-sentence conclusion on whether “older adults show greater sensitivity to outcomes that elicit large absolute prediction errors compared to younger adults”.
13. If I’m correct trial indices (i.e., t subscripts) are missing in equations 1, 5, 6, and 7.
14. It is unclear to me why you would use a transformation of the absolute prediction error instead of the error itself and how this term was derived.

To promote constructive reviewing and transparency, I always sign my reviews,

Jessica Schaaf, PhD
